# Multi-centre classification of functional neurological disorders based on resting-state functional connectivity

**DOI:** 10.1016/j.nicl.2022.103090

**Published:** 2022-06-17

**Authors:** Samantha Weber, Salome Heim, Jonas Richiardi, Dimitri Van De Ville, Tereza Serranová, Robert Jech, Ramesh S. Marapin, Marina A.J. Tijssen, Selma Aybek

**Affiliations:** a Psychosomatic Medicine, Department of Neurology, Inselspital, Bern University Hospital, University of Bern, Switzerland; b Department of Radiology, Lausanne University Hospital and University of Lausanne, Lausanne, Switzerland; c Institute of Bioengineering, École Polytechnique Fédérale de Lausanne, Lausanne, Switzerland; d Department of Radiology and Medical Informatics, Geneva University Hospitals, Geneva, Switzerland; e Centre for Interventional Therapy of Movement Disorders, Department of Neurology, Charles University, 1^st^ Faculty of Medicine and General University Hospital in Prague, Czech Republic; f Department of Neurology, Charles University and General University Hospital in Prague, Prague, Czech Republic; g Department of Neurology, University Medical Center Groningen, Hanzeplein 1, 9713 GZ Groningen, the Netherlands; h UMCG Expertise Center Movement Disorders Groningen, University Medical Center Groningen (UMCG), Groningen, the Netherlands

**Keywords:** Functional connectivity, Biomarker, Multi-site, Conversion disorder, Inter-scanner variability

## Abstract

**Background:**

Patients suffering from functional neurological disorder (FND) experience disabling neurological symptoms not caused by an underlying classical neurological disease (such as stroke or multiple sclerosis). The diagnosis is made based on reliable positive clinical signs, but clinicians often require additional time- and cost consuming medical tests and examinations. Resting-state functional connectivity (RS FC) showed its potential as an imaging-based adjunctive biomarker to help distinguish patients from healthy controls and could represent a “rule-in” procedure to assist in the diagnostic process. However, the use of RS FC depends on its applicability in a multi-centre setting, which is particularly susceptible to inter-scanner variability. The aim of this study was to test the robustness of a classification approach based on RS FC in a multi-centre setting.

**Methods:**

This study aimed to distinguish 86 FND patients from 86 healthy controls acquired in four different centres using a multivariate machine learning approach based on whole-brain resting-state functional connectivity. First, previously published results were replicated in each centre individually (intra-centre cross-validation) and its robustness across inter-scanner variability was assessed by pooling all the data (pooled cross-validation). Second, we evaluated the generalizability of the method by using data from each centre once as a test set, and the data from the remaining centres as a training set (inter-centre cross-validation).

**Results:**

FND patients were successfully distinguished from healthy controls in the replication step (accuracy of 74%) as well as in each individual additional centre (accuracies of 73%, 71% and 70%). The pooled cross validation confirmed that the classifier was robust with an accuracy of 72%. The results survived post-hoc adjustment for anxiety, depression, psychotropic medication intake, and symptom severity. The most discriminant features involved the angular- and supramarginal gyri, sensorimotor cortex, cingular- and insular cortex, and hippocampal regions. The inter-centre validation step did not exceed chance level (accuracy below 50%).

**Conclusions:**

The results demonstrate the applicability of RS FC to correctly distinguish FND patients from healthy controls in different centres and its robustness against inter-scanner variability. In order to generalize its use across different centres and aim for clinical application, future studies should work towards optimization of acquisition parameters and include neurological and psychiatric control groups presenting with similar symptoms.

## Highlights

• Using machine learning on multi-centre data, FND patients were successfully classified with an accuracy of 72%.
• The angular- and supramarginal gyri, cingular- and insular cortex, and the hippocampus were the most discriminant regions.
• To provide diagnostic utility, future studies must include patients with similar symptoms but different diagnoses.

## 1 Introduction

Functional neurological disorders (FND) describe the presence of neurological symptoms not caused by a classical neurological disease (American Psychiatric Association, 2013) but related to brain dysfunctions (Drane et al., 2020). Patients can experience a wide range of neurological symptoms, most frequently motor (e.g., weakness or abnormal movements), sensory (e.g., numbness), or attacks of clouded consciousness which are sometimes accompanied by convulsions (World Health Organization, 1993). Nowadays, the diagnosis of FND is made on the basis of positive clinical signs (Daum et al., 2015, Stone and Carson, 2015), and less emphasis is put on an exclusion process (i.e., not identifying an underlying explanatory neurological disease). Indeed, even if there is no gold standard against which to compare the validity of these signs, several recent studies have shown excellent specificity for several bedside clinical signs (Daum et al., 2015, Espay et al., 2018a, Syed et al., 2011). However, due to heterogeneity of FND symptoms and a broad spectrum of potential differential diagnosis, specialists often request multiple time- and cost-consuming additional tests to rule out an underlying organic lesion or comorbid condition (Espay et al., 2009), even if they were convinced of the diagnosis based on their initial clinical evaluation (Espay et al., 2018a). This highlights the need to identify an adjunctive positive biomarker to support clinicians in their daily clinical routine. Such a marker could allow rapid confirmation of the clinical diagnosis, rather than engaging in a long and exhaustive process of excluding all evoked differential diagnoses.

In the search for new biomarkers in neuropsychiatric disorders, resting-state (RS) functional magnetic resonance imaging (fMRI) has gained growing attention as a promising and easily applicable tool (Greicius, 2008). Resting-state fMRI allows studying blood oxygen dependent level (BOLD) signal fluctuations in the brain under resting condition and therefore does not depend on the patient’s active participation. Furthermore, inter-regional correlations of temporal fluctuations are thought to reflect functional connectivity (FC) between spatially distinct brain regions. Therefore, RS fMRI can reveal important information about underlying neuropathophysiological changes in functional networks of patients (Sokolov et al., 2019, Takamura and Hanakawa, 2017, van den Heuvel and Hulshoff Pol, 2010). Even though task-based fMRI studies are predominant in FND, RS studies in FND were able to confirm findings from task-based studies and identified consistent results. Amongst the existing RS studies, (1) increased limbic connectivity to motor control areas (Baek et al., 2017, Maurer et al., 2016, van der Kruijs et al., 2012), (2) aberrant connectivity from the right temporoparietal junction (TPJ) to sensorimotor regions (Diez et al., 2019, Hassa et al., 2017, Maurer et al., 2016, Mueller et al., 2022, Wegrzyk et al., 2018), as well as (3) altered connectivity from memory-related temporal structures (Longarzo et al., 2020, Monsa et al., 2018, Szaflarski et al., 2018) were identified.

In parallel, the application of machine learning algorithms offers a complementary tool for RS fMRI data analysis. Moreover, machine learning approaches have shown to be robust and sensitive to disease-specific alterations in functional and structural medical images (Erickson et al., 2017). As such, its value has been demonstrated in several neurological diseases and heterogenous psychiatric disorders by successfully distinguishing patients from healthy controls based on RS FC (for review see (Nielsen et al., 2020)).

In the field of FND, our previous study (Wegrzyk et al., 2018) showed promising results with regards to accurately distinguishing FND from healthy controls (HC). We applied a multivariate classification approach based on whole-brain RS FC aiming at discriminating motor FND patients from healthy controls in a predictive setting. Similarly, in another study the seizure-subtype of FND (psychogenic non-epileptic or functional seizures) was successfully classified against healthy controls, based on RS FC (Ding et al., 2013) and T1-weighted structural MRI data (Vasta et al., 2018). Even though real-life use of such a biomarker will need control groups with similar symptoms to FND and not only healthy controls, these studies provided a strong rationale to continue the validation of such classification algorithms. Indeed, most bedside positive signs for FND are specific and reliable, but neuroimaging classification based on machine learning might provide a future clinical tool in the form of an additional rule-in test against other neurological and psychiatric diseases and disorders.

The translation of neuroimaging data from bench to bedside has always been challenging due to the clinical heterogeneity (Espay et al., 2018a, Galli et al., 2020) and within-group differences of neuropsychiatric disorders (i.e., FND patients), and consequently its limited generalizability within and between patient populations (Stone et al., 2011). Importantly, overcoming the problem of low generalizability requires large samples, which includes patients with different symptom types and symptom severities, and preferably from different centres. Furthermore, establishing RS FC as an adjunctive positive biomarker for FND requires its applicability within and across different centres, i.e., different symptom types and symptom severity, consequently increasing the sample size and the heterogeneity of the dataset, which might benefit the classification performance. The next step towards a clinical application therefore includes the validation of multivariate classification approaches in different datasets (i.e., with regard to FND subtypes or scanners), and to assess their performance when using multi-centre data.

To bridge this gap, we set out to further evaluate the classification performance of our previously published classification approach (Wegrzyk et al., 2018) through three different validation steps (Dyrba et al., 2013, Nunes et al., 2020, Rozycki et al., 2018). First, our aim was to replicate the previous results by applying the method in additional datasets collected at other centres (intra-centre cross-validation step) and test its robustness when used in a multi-centre setting by pooling the data of these centres together (pooled cross-validation step). Our second aim was to assess the generalizability of the method by using data from each single centre once as test set after training on the data from the other centres (inter-centre cross-validation step). Successfully distinguishing FND patients from HC in a multi-centre setting could set path towards a clinical application by including neurological and psychiatric controls with similar symptoms (but other diagnoses) in future studies.

## 2 Materials and methods

### 2.1 Participants

Data were collected retrospectively from four different European University Neurology Departments: i) Geneva (Switzerland, previously published in (Wegrzyk et al., 2018)), ii) Bern (Switzerland), iii) Prague (Czech Republic, previously published in (Mueller et al., 2022)) and iv) Groningen (The Netherlands, previously reported in (Marapin et al., 2021, 2020)). Board-certified neurologists confirmed the diagnosis of FND according to DSM-5 (World Health Organization, 1993) and using positive signs (Stone and Carson, 2015).We included FND patients with motor and sensory symptoms (F44.4 and 44.6), with functional seizures (F44.5), and mixed symptom type (F44.7). For movement disorders (F44.4), clinically definite and documented diagnoses according to (Gupta and Lang, 2009) were included. Exclusion criteria were a current neurological disease or disorder (other than FND), alcohol or drug abuse, pregnancy or breast-feeding, and contraindication for MRI scanning. The studies were approved by local ethics committees at each of the centres, i.e., the ethics committee of the University Hospitals of Geneva (CER 14-088), the Competent Ethics Committee of the Canton Bern (SN_2018-00433), the Ethics Committee of the General University Hospital in Prague (approval number 26/15) and the Medical Ethical Committee of the Amsterdam University Medical Center, location AMC, the Netherlands (identification number MEC10/079). All subjects provided written informed consent.

The dataset included 220 MRI scans from patients suffering from FND and age- and sex-matched HC. Data from 21 subjects were excluded due to too high motion artefacts (see section 2.3), and 10 subjects were excluded due to insufficient quality of the functional data (slice artefacts in frontal and/or parietal regions). To maintain an equal number of age- and sex matches, the equivalent age- and sex match of each excluded subject was discarded as well (n = 17), in order to have a well-balanced dataset (Dyrba et al., 2013, Nielsen et al., 2020). We confirmed matched ages within and between the centres using a type III - ANOVA with factor group and centre. The remaining 172 MRI scans included data from 86 patients and their 86 age- and sex-matched healthy controls (Table 2), correspondingly, it needs to be underlined that - as compared to the previous work - two healthy controls were excluded from the original dataset of centre I in order to have equal number of subjects in both groups. Similarly, as compared to the dataset in (Marapin et al., 2021, Marapin et al., 2020), two subjects were excluded due to motion artefacts along with their corresponding age- and sex match).

### 2.2 Data acquisition

Mood disorders are known comorbidities in FND patients (Carson and Lehn, 2016). Therefore, anxiety and depression scores, as well as psychotropic medication (i.e., benzodiazepines, neuroleptics, antidepressants, antiepileptics, and opioids) are commonly assessed in studies on FND patients. Accordingly, centre I, II, and III collected behavioural data of patients and controls on anxiety and depression using the Spielberg State-Trait Anxiety Inventory (STAI, Spielberger et al., 1983) and the Beck’s Depression Inventory (BDI, Beck, 1961). Centre IV collected behavioural data on anxiety and depression in patients using the Beck’s Anxiety Inventory (BAI, Beck et al., 1988) and the Beck’s Depression Inventory (BDI, Beck, 1961). Symptom severity was evaluated using the Clinical Global Impression (CGI) score (0 = no symptoms to 5 = very severe symptoms) in centre I; using the CGI score (0 = no symptoms to 7 = very severe symptoms) in centre II and IV; and using the Simplified Version of the Psychogenic Movement Disorder Rating Scale (S-FMDRS, Nielsen et al., 2017) in centre III. CGI scores with different scales were converted into the same scale. S-FMDRS scores were converted into CGI scores (see Supplementary Material, Appendix 1). Differences in symptom severity between centres (CGI score) were analysed using one-way ANOVA.

Functional and structural MRI data were all acquired on 3-Tesla units using different MRI manufacturers, machines and protocols. Acquisition parameters for the fMRI data of each centre are summarized in Table 1. In one centre (centre IV), fMRI data were based on fast field single echo planar imaging (FEEPI), whereas in the others, it was based on whole-brain single shot multi-slice BOLD echo-planar imaging (EPI). Structural scans were obtained using a T1-weighted Magnetization Prepared Rapid Gradient-Echo (MPRAGE) image in centre I, II, and III; and using a T1 weighted turbo field echo (TFE) image in centre IV.

**Table 1:** Scanner acquisition parameters.

Centre	Model	Manufacturer	TR [s]	TE [ms]	Acquisition time [min]	Volumes	flip angle [°]	Voxel size[mm^3^]
I	Magnetom TrioTim	Siemens	2	20	05:08	150	80	3.0 × 3.0 × 2.5
II	Magnetom Prisma	Siemens	2	20	05:08	150	80	3.0 × 3.0 × 2.5
III	Magnetom Skyra	Siemens	2	30	10:16	300	90	3.0 × 3.0 × 3.0
IV	Philips Intera Medical Systems	Philips	2	30	07:30	225	70	3.5 × 3.5 × 3.5

Abbreviations: TR: repetition time; TE: echo time.

### 2.3 MR pre-processing

Data were pre-processed and analysed using MATLAB (R2017b, MathWorks Inc., Natick, USA). Each centre was pre-processed individually. An adapted version of the previous pre-processing pipeline from (Wegrzyk et al., 2018) based on the Statistical Parametric Mapping version 12 (SPM12) tools (https://www.fil.ion.ucl.ac.uk/spm/software/spm12/) was used, including: functional realignment and co-registration of the mean functional image to the structural image, and segmentation of the structural image into grey matter, white matter, and cerebrospinal fluid. The functional images were additionally checked for excessive head motion using the framewise displacement (FD) method of Power and colleagues (Power et al., 2014). Mean FD and number of volumes above threshold of >0.5 mm were calculated per subject. A type III – ANOVA was used to evaluate differences in motion artefacts for the factors group and centre. Then, for each subject an individual structural brain atlas based on the AAL atlas (Tzourio-Mazoyer et al., 2002) was built using a customized version of the IBASPM toolbox (Aléman-Gomez et al., 2006). From the AAL atlas, we used 88 regions (whole atlas without the cerebellum and pallidum (due to signal drop-out), same as in (Wegrzyk et al., 2018)). The individual structural atlas was mapped onto the native resolution of the functional data. Furthermore, region-averaged time-series were extracted and motion parameters, as well as the average signal from the white matter and the cerebrospinal fluid were regressed out (Richiardi et al., 2011, Wegrzyk et al., 2018). The region-averaged time-series were Winsorized to the 95th percentile to reduce the effect of outliers and linearly detrended. For optimization purposes of the first validation step (see section 2.5), the region-averaged time-courses were either bandpass filtered (0.01–0.08 Hz) or wavelet subband filtered (Richiardi et al., 2011) (see Supplementary Material, Appendix 2 for further details and explanations on the pre-processing pipelines).

### 2.4 Resting-State functional connectivity modelling

Pairwise Pearson correlation coefficients between each pair of atlas regions were calculated for each subject to obtain a correlation matrix (number of regions × number of regions) (Smith et al., 2011). The correlation coefficients were Fisher-Z transformed to make the connectivity matrices Gaussian. The Fisher-Z transformed connectivity matrices of each centre were then connection-wise Z-scored to normalize the data with regard to centre, which acts as a site harmonization. To evaluate the effectiveness of the normalization, we analysed within- and between centre and group effects of functional connectivity differences between each pair of regions using n-way ANOVA before and after normalization. For each subject, the upper triangular part (without the diagonal) of the correlation matrix was extracted and lexicographically organized in a two-dimensional feature representation, which was used further as input feature vectors for the classifier. The feature vector of each subject therefore contained $\left[\left(88\times 87\right)/2\right]=3828$ features. The exact procedure can be found in (Richiardi et al., 2011, Richiardi et al., 2010).

### 2.5 Classification

To perform a binary classification, a Support Vector Machine (SVM) classifier with a linear Kernel function and L2 regularization was used, which learned a discriminative function that separated the two groups as accurately as possible. The SVM implementation for MATLAB of the LIBSVM package (Chang and Lin, 2011) (software available at https://www.csie.ntu.edu.tw/~cjlin/libsvm/) was used, where the C parameter was set at 1. The classification process includes two main steps: 1) training and testing of the model and 2) evaluation of the model. In order to estimate the performance of our model, we chose three cross-validation approaches adapted and similarly implemented as in (Dyrba et al., 2013) and (Nunes et al., 2020):

(1) Intra-centre cross-validation: Each dataset was evaluated individually by separating training and test set by using an *n*-fold leave-one-out (LOO) cross-validation approach, where n represents the number of subjects. For each iteration, *n*-1 subjects were used as training data and the remaining subject was used as test data. This was repeated until each subject within a centre was used once to test the classification performance. During this intra-centre cross-validation, we therefore replicated the results in centre I, and validated its applicability in three other datasets originating from three separate centres (centre II-IV).
(2) Pooled cross-validation: All the data of the four centres were pooled and separated in a training set and a testing set by using the *n*-fold LOO cross-validation approach again. The classifier was trained on n-1 subjects, including all subjects of the four centres, and tested on the remaining subject. This was repeated until each subject from each centre was used once to test the classification performance. During this pooled cross-validation, we evaluated the classifiers performance when working with data that arise from different scanners introducing a scanner-specific variability.
(3) Inter-centre cross-validation: The data from *s-1* scanners were used as a training set and the data from each remaining single centre was used once as a testing set. During this inter-centre cross-validation, we investigated if the learned linear SVM model can be applied to data from an unknown scanner and therefore evaluated its generalization power.

This setting poses great challenges due to the many sources of uncontrolled variance across scanners and datasets (Abraham et al., 2017, Noble et al., 2017). We thus further examined the classification performance when gradually transferring subjects from the test set to the training set. Doing so, the test set is not fully naïve to the potential centre-specific bias introduced in the inter-centre cross-validation setting. This procedure, however, can help to understand the impact of scanner-specific bias to the classification performance. We iteratively transferred data from two subjects (one HC and one FND) from the test set to the training set to examine the learning curve. In each iteration, two more subjects were transferred from the test set to the training set until a maximum number of 28 subjects (i.e., 14 HC, 14 FND) was transferred. Namely, 28 subjects represent the maximum number of subjects that can be transferred in order to have at least two remaining subjects in the test set.

**Fig. 1:**
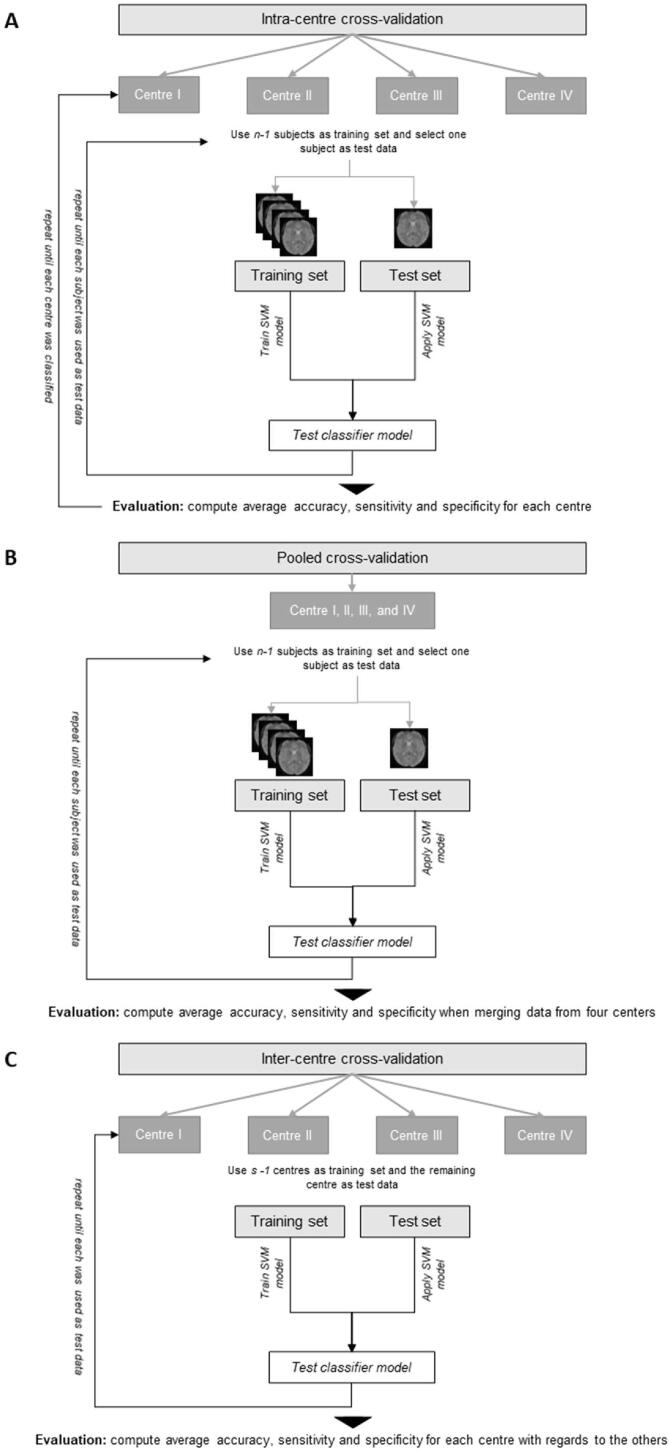
Flow chart of the three cross-validation approaches including (A) intra-centre cross-validation, (B) pooled cross-validation, and (C) inter-centre cross-validation. Throughout the training, a leave-one-out cross-validation (LOOCV) approach was applied.

In each setting, the classification performance was calculated as the average performance across all folds. Fig. 1 gives an overview of the three different validation steps (for a detailed description, see Supplementary Material, Appendix 2).

### 2.6 Evaluation

To evaluate the classifier’s performance, accuracy, sensitivity, specificity, as well as the area under receiver operating characteristic curve (AUC) were computed. The accuracy provides information about the overall performance of the classifier with respect to both groups and was defined as accuracy = (TP + TN)/n where TP is the number of true positives (patients correctly classified as patients), and TN is the number of true negatives (controls correctly classified as controls) and n is the total number of subjects. The sensitivity is the true positive rate and the specificity the true negative rate, i.e., sensitivity = TP/(TP + FN), specificity = TN/(TN + FP), where FN and FP refer to the number of false negatives and false positives, respectively. The AUC assesses the probability of correctly classifying a random pair of patient and control. It reflects test accuracies as follows: AUC = 1 refers to perfect accuracy, AUC between 0.7 and 0.9 refers to moderate, AUC between 0.5 and 0.7 = refers to low and, AUC = 0.5 is uninformative. To assess the significance of the classification, we performed permutation testing, i.e., the classification was repeated 1000 times using its null distribution with the group labels (patients/control) randomly permuted.

### 2.7 Post-hoc analyses

#### 2.7.1 Most discriminative connections

To shed light on which brain areas may be linked to the pathophysiology of FND and common across all four centres, we focussed the post-hoc analyses on the validation steps which pooled all the data from the four centres (step 2: pooled cross-validation). In order to explore the connections that were most discriminative to distinguish patients and controls, we analysed the highest weights assigned by the classifier to the different functional connections (i.e., correlation coefficients).

Within these most discriminative connections, we then further identified those regions that appeared with the highest frequency. From this set of regions, we analysed the connectivity differences between patients and controls by exploring whether these regions were hypo- or hyper-connected in patients versus controls. For this purpose, we calculated the mean connectivity between the corresponding pairs of regions for each group (healthy controls and FND patients).

### 2.8 Impact of anxiety, depression, medication, and clinical score on classification performance

In order to verify that our results were not driven by potential confounding factors like anxiety (STAI), depression (BDI), psychotropic medication (yes/no), and clinical scores/symptom severity (CGI), we used a logistic regression analysis (using *glm* function in R, which automatically removes missing data from regression analysis). Specifically, we test whether the aforementioned factors could predict if a subject was classified correctly or not (yes/no). We tested each factor individually and in combination.

## 3 Results

### 3.1 Demographic and clinical data

Data from 86 FND patients and 86 age- and sex-matched healthy controls, arising from four different centres were included in this study. All patients and 71 HC completed the Beck’s Depression Inventory (BDI, (Beck, 1961)); 71 patients and 71 HC completed the State-Trait Anxiety Inventory (STAI-S, (Spielberger et al., 1983)). Two patients of centre II were not rated using CGI. Demographic and clinical data are presented in Table 2. There was no significant difference in age between centres and groups. One-way ANOVA on symptom severity (CGI scores) identified a significant effect of factor centre. Post-hoc Tukey's honestly significant difference (Tukey’s HSD) showed that the difference in symptom severity between centre I and IV (p = 0.02), between centre II and IV (p = 0.001) and centre III and IV (p = 0.011) were statistically significant, meaning centre IV had more severe cases than the three other centres.

**Table 2:** Demographic and clinical characteristics of the four centres.

	Centre I		Centre II		Centre III		Centre IV	
	FND (n = 23)	HC (n = 23)	FND (n = 24)	HC (n = 24)	FND (n = 24)	HC (n = 24)	FND (n = 15)	HC (n = 15)
Age, mean (SD), years	42.4 (13.9)	41.8 (13.3)	39.8 (13.2)	35.5 (13.3)	42.6 (10.6)	44.3 (9.41)	40.8 (12.2)	40.7 (13.2)
Sex (females/males)	21/2	20/3	14/10	16/8	21/3	21/3	7/8	8/7
Disease severity (CGI, median, quantile)	2 [0.5–3]	*NA*	1 [1–2]	*NA*	1 [1–2]	*NA*	3 [2–3]	*NA*
Psychotropic medicament intake (yes/no)	14/9	0/23	6/18	1/23	11/13	7/17	*NA*	*NA*
Symptom type^a^	12 weakness 4 seizures 2 gait disorder 5 dystonia 7 tremor 1 myoclonus	*NA*	11 weakness 3 seizures 12 gait disorder 1 dystonia 7 tremor 2 myoclonus	*NA*	18 weakness 1 seizures 4 gait disorder 2 dystonia 9 tremor 1 myoclonus	*NA*	3 tremor 13 myoclonus	*NA*
BDI score, mean (SD)	11.3 (5.18)	6.44 (6.27)	11.0 (11.7)	3.54 (3.82)	18.0 (14.9)	11.8 (13.1)	8.33 (8.41)	*NA*
STAI-S score, mean (SD)	60.6 (13.8)	60.7 (15.1)	73.5 (23.0)	64.5 (17.1)	90.7 (28.4)	84.0 (22.7)	*NA*	*NA*
BAI score, mean (SD)	*NA*	*NA*	*NA*	*NA*	*NA*	*NA*	17.2 (13.3)	*NA*

Data from centres I and IV are not the exact same data as used in the previous publications, due to the exact age- and sex match. Abbreviations: *FND*: functional neurological disorders, *HC*: healthy controls, *BDI*: Beck's Depression Inventors, *STAI*: State-Trait Anxiety Inventory, *CGI*: Clinical Global Impression Score ranging from 0 = none, 1 = mild, 2 = moderate, 3 = severe, 4 = very severe, *SD* = standard deviation, *NA* = not applicable.

a Patients can present with more than one symptom type.

FND symptom type was similar between centre I to III with a majority of abnormal movement (F44.4) diagnosis (see Table 2 for details) as well as functional seizures (F44.5) or mixed (F44.7) whereas centre IV had exclusively abnormal movements (F44.4) cases.

### 3.2 Framewise displacement

FD measures showed a significant main effect of *centre* (F(3,164) = 5.5210, p = 0.001). Post-hoc multiple comparison of means showed that the difference between centre I and centre III (p < 0.0001) and centre IV (p = 0.0006), as well as between centre II and centre III (p = 0.0002) and IV (p = 0.008) were statistically significant (Supplementary Material, Figure S1), meaning that centres III and IV had more motion artefacts as compared to centre I and II.

### 3.3 Replication and robustness of classification approach

(1) *Replication:* Applying the method from (Wegrzyk et al., 2018) on the slightly modified sample size (see section 2.1) found very similar values: accuracy of 73.9 % (published 68.8%), as well as a highly balanced sensitivity of 69.6% (published 68%), specificity of 78.3% (published 69.6%), and with AUC of 0.86.
(2) *Intra-centre cross-validation*: The exact same method, when applied to centres II, III and IV, yielded accuracies ranging from 70 to 72.9% (p = 0.02–0.001). Equivalently, the sensitivity and specificity were balanced (sensitivity: 70.8–79.2%, specificity: 66.7–70.8), and their diagnostic abilities - indicated by the AUC - were moderate to good in all three centres (see Table 3 for details).

**Table 3:** Classification performance of the intra-centre and pooled validation steps on the four different centres.

Centre	Accuracy (%)	Specificity (%)	Sensitivity (%)	AUC	p-value
***Intra-centre cross-validation***					
I	73.9	78.3	69.6	0.86	0.001
II	72.9	66.7	79.2	0.73	0.002
III	70.8	70.8	70.8	0.67	0.002
IV	70	66.7	73.3	0.75	0.02

***Pooled cross-validation***					
	71.5	75.6	67.4	0.79	0.003
(3) *Pooled cross-validation*: When data from the four centres were pooled, a significant classification accuracy of 71.5% (sensitivity: 67.4%, specificity 75.6%, AUC: 0.79, p = 0.003, see Table 3 for details) was found. We present below the list of most discriminative features with their SVM weights, the confusion matrix, and the receiver operating characteristic (ROC) curve and of the pooled cross-validation in Fig. 2.

**Fig. 2:**
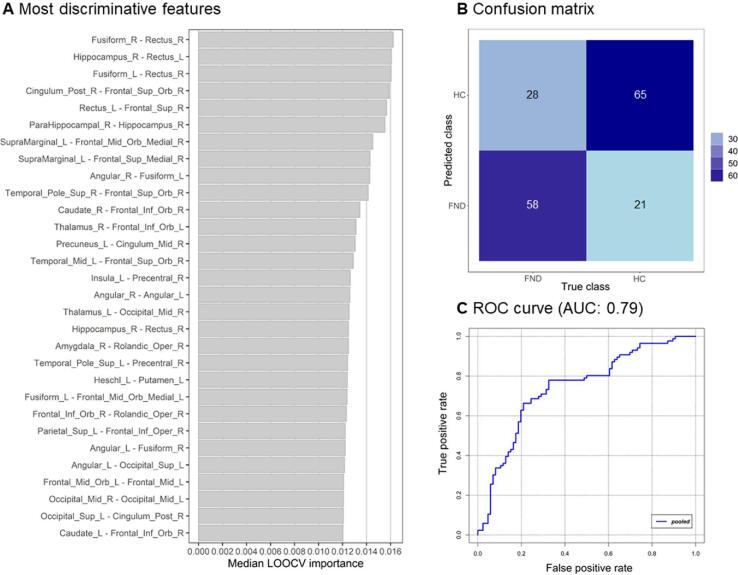
Classification results of the pooled cross-validation, showing in (A) on overview over the 30 most discriminative features to distinguish FND from HC representing the weights assigned by the classifier (Median LOOCV importance). LOOCV refers to leave-one-out cross-validation. (B) confusion matrix for the pooled cross-validation, and (C) the receiver operating characteristics (ROC) curve, and area-under-the-curve (AUC) for the pooled cross-validation.

A visual representation of accuracy, sensitivity, specificity across all centres, and ROC curve of the intra-centre- and inter-centre cross-validation can be found in Supplementary Material, Figure S2/S3.

### 3.4 Post-hoc analyses

#### 3.4.1 Most discriminative connections

In the pooled cross-validation, regions such as the hippocampus, the bilateral angular gyrus, the cingulate cortex, bilateral frontal regions and the bilateral supramarginal gyrus were most frequently found within the most discriminative connections. When exploring the connectivity differences between patients and controls in the regions yielding the most discriminative connections, we identified increased connectivity in patients between:

(a) the hippocampus and temporal regions (e.g., right superior temporal gyrus and middle temporal pole), the cingulate cortex, and the bilateral precuneus
(b) the bilateral angular gyrus and sensorimotor regions (e.g., postcentral gyrus), the bilateral fusiform gyrus, and the left superior occipital gyrus
(c) right cingulate cortex and right frontal regions (e.g., orbitofrontal gyrus) and the right thalamus

Similarly, we identified decreased connectivity in patients between.

(a) the right hippocampus and right frontal regions (e.g., inferior orbitofrontal gyrus), subcortical regions (e.g., bilateral parahippocampal gyrus and bilateral amygdala) and subcortical structures (left putamen)
(b) the anterior cingulate cortex and the right caudate
(c) the right and left amygdala
(d) left supramarginal gyrus and frontal regions (e.g., orbitofrontal and middle frontal gyrus)

**Fig. 3:**
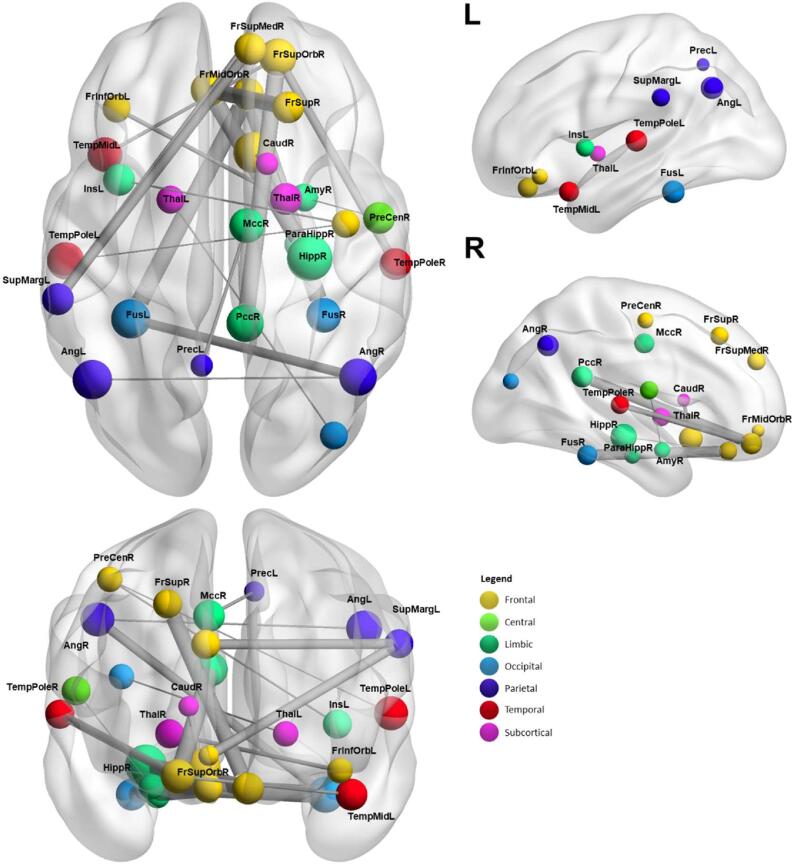
Regions yielding the most discriminative connections of the pooled classification based on the AAL atlas. Size of the nodes correspond to nodal degree, respectively occurrence within the most discriminative connections. Colour of the nodes corresponds to different lobes of the AAL. Thickness of edges correspond to SVM weights. Thicker edges therefore indicate higher SVM weights, respectively higher discrimination power. The mean functional connectivity values corresponding to this figure can be found in Supplementary Material, Table S1. The figures corresponding to each single centre can be found in Supplementary Material, Figure S5.

For visualization purposes, regions yielding the most discriminative connections for the pooled cross-validation are presented in Fig. 3 (the corresponding figure for each single centre can be found in Supplementary Material, Figure S5). A figure displaying hyper- and hypoconnectivity between the regions yielding the most discriminative connections can be found in Supplementary Material, Figure S4. Data were visualized using BrainNet Viewer (Xia et al., 2013). Mean functional connectivity in controls and patients between pairs of regions showing most discriminative functional connectivity of the pooled cross-validation can be found in the Supplementary Material, Table S1).

#### 3.4.2 Logistic regression of anxiety, depression, medication, and clinical scores

Whether a subject was classified correctly or not (yes/no) could not be predicted by anxiety, depression, medication and clinical scores - neither in the intra-centre nor in the pooled cross-validation setting (Supplementary Material, Table S2). These potential confounding factors thus did not drive the classification performances.

### 3.5 Generalizability to multi-centre data

(1) *Inter-centre cross-validation*: When data from each single centre were used once to test the classifier and data from the remaining three centres were used to train the classifier, we found classification accuracies ranging from 37.5 to 50% (sensitivity: 37.5 – 56.5%, specificity: 33.3 – 54.2%), below chance level. Correspondingly, the AUC was below chance (see Table 4 for details).

**Table 4:** Classification performance of the inter-centre cross-validation step on the four different centres.

***Inter-centre cross-validation***					
Centre	Accuracy (%)	Specificity (%)	Sensitivity (%)	AUC	p-value
Test set: I	50	43.5	56.5	0.46	0.1
Test set: II	37.5	33.3	41.7	0.43	1.0
Test set: III	45.8	54.2	37.5	0.41	1.0
Test set: IV	46.7	46.7	46.7	0.48	1.0
(2) *Centre normalization of functional connectivity data*: After normalization (see section 2.3), n-way ANOVA on the different connections with factor *group* and *centre*, corrected for multiple comparisons using false discovery rate (FDR), showed only a significant effect of factor group in 287 connections. No centre nor interaction effect was found. After normalization, functional connectivity thus only differed between groups (FND and HC), but no centre effect remained.
(3) *Adapting the inter-centre cross-validation:* By gradually transferring two subjects (1 HC and 1 FND) from the test set to the training set, we observed an improvement of the overall classification performance to the level of the intra-centre and pooled cross-validation. However, after the transfer of approximately 16–20 subjects, the model started overfitting the results. The different learning curves of accuracy, sensitivity, and specificity of the four centres are presented in the Supplementary Material, Figure S4).

## 4 Discussion

### 4.1 Classification

In line with our first aim, these results show that classification of RS fMRI brain images with a machine learning algorithm (Wegrzyk et al., 2018) could be successfully replicated in three separate samples stemming from different recruiting centres. This means that, overall, this method can successfully distinguish FND patients from healthy controls with accuracies at or above 70% (centre I: 73.9%/II: 72.9%/III: 70.8%/IV: 70.0%). Importantly, these results confirm that the method provides an accurate and robust classification of FND patients and healthy controls within different MRI scanners – as the four centres had different manufacturers and acquisition parameters – when the models are trained at each site. It also shows robustness against clinical heterogeneity, because the FND populations of the four centres were not identical in terms of symptom type and severity. Namely, centre IV included only functional movement disorders (F44.4), whereas centre I to III included mixed (F44.7) cohorts. Patients included in centre IV rated their symptoms as more severe compared to the FND patients included in the other centres.

To strengthen this first validation step, we examined if the classification approach is also robust when merging the data from all four centres together. Therefore, we ran the exact same analysis in a second validation step by pooling all the data together, this yielded a similarly high classification accuracy of 71.5%. Similar results have been found among diverse neurological and psychiatric conditions (for review: Nielsen et al., 2020, Orrù et al., 2012). This strongly suggests that machine learning is an appropriate and robust tool to detect differences in functional connectivity in FND patients and HC. Furthermore, despite the clinical heterogeneity and potential inter-centre confounding factors (e.g., inter-scanner variability), the classifier yielded high classification accuracies. Using a post-hoc logistic regression analysis, we could additionally show that neither anxiety, depression, psychotropic medication intake, nor clinical scores had an impact on classification performance. These results indicate that our model probably discriminated between patients and controls based on features specific to the underlying FND pathology (i.e., aberrant functional connectivity) and not the clinical comorbidities, nor the symptom severity of FND patients. The underlying changes in functional connectivity – independent of symptom type and severity - might represent a FND specific trait, rather than a state. To further verify what these FND specific traits are, however, it is of utmost importance to compare the classification performance against other patient groups with similar symptoms but different diagnoses (e.g., other neurological disorders and/or psychiatric controls). Moreover, it must be considered that other predisposing factors might potentially drive the classification performance. Namely, the aetiology of FND is multifactorial. For instance, genetic risk factors or preceding traumatic life events are thought to affect the pathophysiological mechanisms of FND (Hallett et al., 2022). Particularly, traumatic life experiences and childhood adversities are known risk factors with average odds ratio between 2 and 4 (Ludwig et al., 2018). Moreover, functional and structural alterations have been detected in FND patients in the context of trauma exposure, particularly in regions pointed out by the pooled analysis such as the cingulate cortex, insula, and the hippocampus (Aybek et al., 2015, Aybek et al., 2014, Diez et al., 2020, Maurer et al., 2016, Perez et al., 2017). To the best of our knowledge, this is the first study using multi-centre data of FND patients including different symptom types and symptom severity for a multivariate classification approach. Moreover, machine learning algorithms seem to be robust enough against different symptom types and severity scores, as represented in our results.

In line with our second aim, we evaluated the generalizability of this classification approach by examining whether data from a naïve centre can be correctly classified when applying a model that has been trained on data from the three other centres. Even though we normalized with respect to centre, this third validation step showed that individual classification accuracies did not exceed chance level. Compared to the pooled validation, this step introduced scanner bias of the left-out centre only during the testing, whereas during the pooled cross-validation setting the scanner bias was already included in the training set. This suggests that variance introduced by inter-scanner variability is too high to be overcome using inter-centre cross-validation and might be substantially different from variance introduced by other confounding factors such as comorbidities or symptom severity. With our post-hoc adaptation of the inter-centre cross-validation setting, in which we gradually transferred subjects from the test set to the training set in order to introduce centre-specific scanner bias already during the training, we observed a gradual increase in overall classification performance. This observation strengthens our assumption of that inter-scanner variability plays a critical role and cannot be overcome in our inter-centre cross-validation setting. Indeed, inter-scanner variability is a well-known bias for multi-centre RS fMRI data (Noble et al., 2017, Zhao et al., 2018) that yet has to be overcome. Specifically for multi-centric fMRI graph data, not only functional, but also structural imaging data has been shown to influence graph representation, as fMRI data is parcellated according to the structural MRI data (Castrillon et al., 2015). Neither did regressing out the site substantially aid the classification (Castrillon et al., 2015). Alternatively, our sample size might be too small to properly capture sufficient variation within each site (whether subject-driven or related to technical factors) to generalize to completely unseen sites. Another study on multi-site resting-state connectivity classification for Autism spectrum disorder showed that, given sufficient subjects in the training set (between 280 and 500 depending on inclusion criteria), inter-site performance could reach intra-site performance, but that this was not the case at smaller sample sizes (Abraham et al., 2017). The assumption that a sample size may be too small, can be strengthened by the fact that after normalizing the data, no significant centre effect remained.

In summary, a multi-centre scenario increases the sample size (i.e., in our second validation step) and consequently the heterogeneity of the sample, which might benefit the classification performance. On the contrary, it introduces systematic inter-scanner variability (“site bias”) which is unrelated to the underlying disorder of interest and thus might complicate the discriminative power (Abdulkadir et al., 2011). Consequently, there are only a few studies investigating the applicability of multi-centre classification based on RS FC. In line with our findings, equivalently good classification performances were achieved in pooled multi-centre classification settings using a SVM classifier based on RS FC e.g., for autism spectrum disorder (N = 240 subjects, accuracy = 79%; Chen et al., 2016), for mild cognitive impairment (N = 367 subjects, accuracy = 72%;Teipel et al., 2017), as well as for major depressive disorder (N = 358 subjects, accuracy = 73%; Nakano et al., 2020). The latter also investigated robustness against site bias on classification using a leave-one-site-out cross-validation (LOSO-CV; equivalent to our inter-centre cross-validation). Comparable with our results, their LOSO-CV did not succeed in classifying major depressive disorder in a fully unknown dataset.

The inter-scanner variability clearly limited the classification performance and generalizability when data from a specific scanner was only used for testing but not during the training. Combining data from different modalities, has been found to be one solution to overcome the limitations of multi-centre RS fMRI (Zhuang et al., 2019). For instance, high classification accuracies were achieved in pooled as well as LOSO-CV combining T1-weighted (structural/anatomical) images with RS functional connectivity from patients with frontotemporal dementia and healthy controls (Donnelly-Kehoe et al., 2019). Accordingly, the successful classification of functional seizures based on structural imaging data (Vasta et al., 2018) would suggest employing multi-modal data of FND patients for future classification approaches when working towards a clinical application. Furthermore, previous studies attempted to identify and characterize inter-scanner variability and how they influence fMRI data (Dansereau et al., 2017, Friedman et al., 2006). As such, classification was found to be improved by site harmonization methods (Nakano et al., 2020, Yamashita et al., 2019, Yu et al., 2018). Site harmonization approaches, however, still face methodological challenges: Recent studies raised concerns that site harmonization methods might interfere with analytical methods (Chen et al., 2022), depend on choice of atlas (Yu et al., 2018), or can be substantially impacted by the use of fMRI acquisition parameters (Mori et al., 2018, Yamashita et al., 2019). Apart from using site harmonization approaches, promising results have also been found when applying unsupervised machine learning algorithms such as deep learning. Although they are computationally more complex, they appeared to be robust against site differences (Dewey et al., 2019, Zeng et al., 2018). At last, a feature selection could be implemented in order to reduce the high dimensionality of our feature vectors (Guyon et al., 2003). However, the aim of this project was to examine the generalizability of the previously applied method on different movement disorders/FND centres, rather than developing the best possible machine learning approach suitable for a multi-centre setting. Nevertheless, this could be the goal of future additional validation studies.

### 4.2 Connectivity patterns

Upon visualization of the most discriminative weights of individual connections, we could evaluate their individual contribution to the overall classification. Our study identified regions as most discriminative that indeed were commonly reported in the literature, such as the cingulate cortex (Aybek et al., 2015, Baek et al., 2017, Blakemore et al., 2016, Marapin et al., 2020), right temporal regions (i.e., the temporoparietal junction, TPJ) (Aybek et al., 2014, Espay et al., 2018b, Maurer et al., 2016), the amygdala (Aybek et al., 2015, Morris et al., 2017, Voon et al., 2011), the insula (Espay et al., 2018b, Stone et al., 2007, Voon et al., 2011), the inferior frontal gyrus (IFG, Espay et al., 2018b) or the dorsolateral prefrontal cortex (dlPFC, Aybek et al., 2014, Voon et al., 2016, Voon et al., 2011). However, feature weights need to be interpreted with caution, as a machine learning algorithm values the utility for classification, rather than the clinical relevance of a feature (Nielsen et al., 2020, Nunes et al., 2020). Therefore, one should not infer upon the potential underlying mechanisms of a disorder, but rather examine the weights for their potential pathophysiological validity. As such, our results provided connectivity patterns that are particularly interesting to further construe: connections including 1) the angular- and supramarginal gyri, to sensorimotor regions and 2) cingular- and insular cortex, to hippocampal regions. The angular and supramarginal gyrus are located within/bordering the temporo-parietal junction (TPJ), a key structure for FND. Abnormal interaction between the TPJ and sensorimotor regions has been repeatedly found in FND patient and is thought to be associated with their impaired sensory prediction signal (i.e., the sense of agency) (Perez et al., 2012, Voon et al., 2010). Similarly, RS-fMRI study in FND identified decreased connectivity from the TPJ to sensorimotor regions (Maurer et al., 2016), to the precuneus (Mueller et al., 2022), and between the TPJ, motor regions, cingulate cortex and insula (Diez et al., 2019), as well as decreased connectivity between the right inferior parietal cortex to the dlPFC and the anterior cingulate cortex (Baek et al., 2017) supporting the theory of impaired sensorimotor integration and impaired sense of agency. On the other hand, the cingular- and insular cortex, and hippocampal regions belong – amongst others - to the limbic network and are considered to be part of the emotion-cognition integrative system (Pessoa, 2008). Altered connectivity in FND in limbic regions have been associated with abnormal frontal lobe emotional control and emotion-motion interactions (Aybek et al., 2014, Monsa et al., 2018). In particular, aberrant hippocampus activity was found in response to aversive stimuli in task-based fMRI using emotional stimuli (Aybek et al., 2014, Blakemore et al., 2016, Szaflarski et al., 2018). Moreover, increased FC was found between the cingulate cortex, precuneus, and the ventromedial prefrontal cortex during a motor task (Cojan et al., 2009). Similarly, RS fMRI studies on FND identified increased connectivity from parahippocampal structures to the right superior temporal gyrus (Longarzo et al., 2020) and to the middle- and inferior temporal gyrus (Szaflarski et al., 2018), increased connectivity between the hippocampus and default mode network (DMN) related regions (Monsa et al., 2018), as well as increased FC from the amygdala to the dlPFC (Morris et al., 2017). Alterations in RS FC in these regions thus support previous findings on task-based fMRI stating an impaired emotion regulation in FND (Aybek et al., 2015, Aybek et al., 2014, Espay et al., 2018b).

### 4.3 Towards a clinical application

Excellent sensitivity and specificity (between 80 and 100%) has been found for bedside clinical signs (Daum et al., 2015, Espay et al., 2018a, Syed et al., 2011). However, these maneuvers may still face several limitations, including a lack of gold standards against which to compare them and unblinded assessments in most studies along with other methodological issues such as a poor description of how the diagnosis of FND was made. Additional diagnostic procedures might support the clinical diagnostic process. With regard to a multivariate classification approach applied within a clinical setting, an accuracy of 70% might not present a final solution. The setting of classifying patients against healthy controls does not represent the clinical need and limits the generalizability of these results to clinical application at this stage. For daily clinical routine, one should rather aim at distinguishing a functional symptom from identical/similar neurological and psychiatric symptoms, and not from a healthy control. The potential applicability of such a machine learning approach would be for example to assist screening of patients in the emergency department in cases of ambiguous neurological symptoms or could provide more details in difficult cases. Therefore, rather than replacing a clinical diagnosis, it might provide additional diagnostic support in the form of additional rule-in tests. A patient with a functional disorder could easier be identified as such - in addition to the bedside clinical signs - and could be directly referred to a specialist, before undergoing multiple medical tests and examinations (Espay et al., 2009). Besides, the medico-legal context highlights the importance of identifying an adjunctive positive biomarker in order to help distinguishing FND from intentionally produced neurological symptoms as observed in malingering or factitious disorders in which patients fabricate their symptoms or simply are feigning or lying about their symptoms (Colombari et al., 2021). Therefore, to test the power against differential diagnoses, it is of utmost importance – as a next step - to classify FND patients against similar psychiatric patients, trauma patients or against neurological patients with the same or similar symptoms (e.g., dystonia, essential tremor, Parkinson’s disease, or multiple sclerosis). In summary, machine learning algorithms could thus further support differential diagnoses and optimize treatment prevention and patient management. However, diagnostic utility is only provided if these results can be replicated in other patients with the same or similar symptoms, but different diagnoses.

### 4.4 Limitations and future directions

This study has several limitations. Even though data from four different centres were used, the sample size is small compared to other multi-centre classification studies using multi-centre data bases, such as the Alzheimer’s Disease Neuroimaging Initiative (ADNI) (Jack et al., 2008) or the Autism Brain Imaging Data Exchange (ABIDE) project (Di Martino et al., 2014). To date, a large, multi-centre database sharing imaging data of FND patients unfortunately does not exist. Small sample sizes have been associated with higher reported accuracies without properly controlling for overfitting (Vabalas et al., 2019). We avoided overfitting by perfectly matching our groups within- and between centres and by applying a leave-one-out cross-validation approach, which is a powerful tool against overfitting and recommended in small samples (Vabalas et al., 2019). Accordingly, our results of the intra-centre and pooled cross-validation are comparatively high with significant accuracies and highly balanced sensitivities and specificities. Nevertheless, a multi-centre database would bring the advantage of adjusting scanner protocols on each centre and scanner type and would thus provide comparably high data quality and low inter-scanner variability. Thereby, multi-centre imaging studies must be planned carefully with regards to scanner hardware and software, implementation of an appropriate quality assurance program to properly validate and monitor data, and application of proper site standardization methods (for recommendations see Glover et al., 2012).

A second limitation is the use of only one atlas with 90 cortical- and subcortical regions. As for now, the purpose of this project was to validate the previously published method across different centres, no changes were made to the pre-processing pipeline. Despite involving a higher computational load, a more fine-grained parcellation (e.g., Glasser atlas (Glasser et al., 2016)) or a voxel-wise approach could detect different information (Eickhoff et al., 2018), and may aid the future development of an adjunctive imaging-based biomarker. On the contrary, using an approach with a higher spatial resolution also bears the risk of overfitting or missing important information due to the comparable high amount of probably uninformative features (Erickson et al., 2017).

A third limitation is that centres III and IV were found to have higher head motion than centres I and II, what might negatively affect functional connectivity (Van Dijk et al., 2012). The significant results obtained in intra-centre and pooled-centre validation, however, indicate that even patients known to have a lot of movements (Centre III and IV had more motor subtypes of FND F44.4) can be correctly classified. For future studies, subjects should be strictly advised to lay calmly, and their head should be fixed using foam cushions. Ideally, prospective motion correction techniques including motion-tracking cameras or a pilot tone approach (Ludwig et al., 2021) could be used to further improve data quality in this respect.

A last limitation is that clinical data where not uniformly collected and used different scales (CGI, S-FMDRS scales), which meant that scales needed to be adjusted. Including symptom severity in our post-hoc logistic regression analysis is therefore not optimal, as the transformation we have done from S-FMDRS to CGI is intuitive but not validated. Similarly, as anxiety and/or depression scores were collected using different questionnaires (STAI, BDI or BAI), the regression analysis showing no influence of mood on the classification performance should be interpreted with caution until future studies confirm this with prospectively collected uniform clinical data. Together with the uneven distribution of symptom types, we cannot fully account for it with good reliability. From a technical point of view, a future project should aim at balancing the different symptom types, so that a data-driven machine learning approach would learn to recognize those patients as well who are normally underrepresented in a clinical setting. To overcome the problem of different symptom type distribution, patients could also be stratified according to their symptom types and/or include the clinical data (e.g., CGI) into the model (Patel et al., 2015). In order to achieve this in a multi-centre setting, it would be necessary that the same clinical data and psychiatric comorbidities are collected using the same clinical scores and identical questionnaires in each centre. Additionally, data on traumatic life events or childhood adversities should be collected, in order to assess the potential influence on functional brain aberrancies.

## 5 Conclusion

In summary, multi-centre RS FC has shown its potential to distinguish FND patients from HC. These findings set the ground for future research on adjunctive biomarkers for FND as the method will need to be improved regarding its generalizability regarding inter-scanner variability and the heterogeneity of symptoms, comorbidities, and severity of symptoms. To provide diagnostic utility, future studies must investigate the classification power when classifying FND patients against classical neurological diseases and/or psychiatric disorders as this would represent a closer setting to the clinical daily routine and could be used as a decision support method for the clinical diagnosis. Importantly, not to replace the clinical diagnosis, but to provide additional rule-in criteria for the diagnosis instead.

## Funding/support

This work was supported by the Swiss National Science Foundation (SNF Grant PP00P3_176985 for SA) and the Leenaards Nested Project grant (Grant 3642 for SA). TS was supported by the Czech Ministry of Health Projects AZV NU20-04-0332 and AZV NV19-04-00233, and by the General University Hospital in Prague MH CZ-DRO-VFN64165. MAJT is funded by ZonMWTOP, European Fund for Regional Development, Provincie Fryslân, Stichting Wetenschapsfonds Dystonie Vereniging, and unrestricted educational grant from Merz.

### CRediT authorship contribution statement

**Samantha Weber:** Conceptualization, Methodology, Software, Formal analysis, Investigation, Writing – original draft, Writing – review & editing, Visualization, Project administration. **Salome Heim:** Project administration, Investigation. **Jonas Richiardi:** Conceptualization, Methodology, Software, Writing – review & editing. **Dimitri Van De Ville:** Conceptualization, Methodology, Software, Writing – review & editing. **Tereza Serranová:** Investigation, Writing – review & editing. **Robert Jech:** Investigation. **Ramesh S. Marapin:** Investigation, Writing – review & editing. **Marina A.J. Tijssen:** Investigation. **Selma Aybek:** Conceptualization, Resources, Writing – review & editing, Supervision, Funding acquisition.

## Declaration of Competing Interest

The authors declare that they have no known competing financial interests or personal relationships that could have appeared to influence the work reported in this paper.

## References

[b0005] Abdulkadir A., Mortamet B., Vemuri P., Jack C.R., Krueger G., Klöppel S. (2011). Effects of hardware heterogeneity on the performance of SVM Alzheimer’s disease classifier. Neuroimage.

[b0010] Abraham A., Milham M.P., Di Martino A., Craddock R.C., Samaras D., Thirion B., Varoquaux G. (2017). Deriving reproducible biomarkers from multi-site resting-state data: an Autism-based example. Neuroimage.

[b0015] Aléman-Gomez Y.-M.-G., Melie-Garcia L., Valdés-Hernandez P. (2006).

[b0020] American Psychiatric Association (2013). Diagnostic and statistical manual of mental disorders. American Psychiatric Association.

[b0025] Aybek S., Nicholson T.R., O’Daly O., Zelaya F., Kanaan R.A., David A.S., Park S. (2015). Emotion-motion interactions in conversion disorder: an fMRI study. PLoS One.

[b0030] Aybek S., Nicholson T.R., Zelaya F., O’Daly O.G., Craig T.J., David A.S., Kanaan R.A., O’Daly O.G., Craig T.J., David A.S., Kanaan R.A. (2014). Neural correlates of recall of life events in conversion disorder. JAMA Psychiatry.

[b0035] Baek K., Doñamayor N., Morris L.S., Strelchuk D., Mitchell S., Mikheenko Y., Yeoh S.Y., Phillips W., Zandi M., Jenaway A., Walsh C., Voon V. (2017). Impaired awareness of motor intention in functional neurological disorder: implications for voluntary and functional movement. Psychol. Med..

[b0040] Beck A.T. (1961). An inventory for measuring depression. Arch. Gen. Psychiatry.

[b0045] Beck A.T., Epstein N., Brown G., Steer R.A. (1988). An inventory for measuring clinical anxiety: psychometric properties. J. Consult. Clin. Psychol..

[b0050] Blakemore R.L., Sinanaj I., Galli S., Aybek S., Vuilleumier P. (2016). Aversive stimuli exacerbate defensive motor behaviour in motor conversion disorder. Neuropsychologia.

[b0055] Carson A., Lehn A. (2016). Epidemiology. Handb. Clin. Neurol..

[b0060] Castrillon, J.G., Ahmadi, A., Navab, N., Richiardi, J., 2015. Learning with multi-site fMRI graph data. Conf. Rec. - Asilomar Conf. Signals, Syst. Comput. 2015-April, 608–612. https://doi.org/10.1109/ACSSC.2014.7094518.

[b0065] Chang C.-C., Lin C.-J. (2011). LIBSVM: a Library for support vector machines. ACM Trans. Intell. Syst. Technol..

[b0070] Chen A.A., Srinivasan D., Pomponio R., Fan Y., Nasrallah I.M., Resnick S.M., Beason-Held L.L., Davatzikos C., Satterthwaite T.D., Bassett D.S., Shinohara R.T., Shou H. (2022). Harmonizing functional connectivity reduces scanner effects in community detection. Neuroimage.

[b0075] Chen H.H., Duan X., Liu F., Lu F., Ma X., Zhang Y., Uddin L.Q., Chen H.H. (2016). Multivariate classification of autism spectrum disorder using frequency-specific resting-state functional connectivity—A multi-center study. Prog. Neuro-Psychopharmacology Biol. Psychiatry.

[b0080] Cojan Y., Waber L., Carruzzo A., Vuilleumier P. (2009). Motor inhibition in hysterical conversion paralysis. Neuroimage.

[b0085] Colombari M., Di Vico I.A., Turrina S., De Leo D., Tinazzi M. (2021). Medico-legal aspects of functional neurological disorders: time for an interdisciplinary dialogue. Neurol. Sci..

[b0090] Dansereau C., Benhajali Y., Risterucci C., Pich E.M., Orban P., Arnold D., Bellec P. (2017). Statistical power and prediction accuracy in multisite resting-state fMRI connectivity. Neuroimage.

[b0095] Daum C., Gheorghita F., Spatola M., Stojanova V., Medlin F., Vingerhoets F., Berney A., Gholam-Rezaee M., Maccaferri G.E., Hubschmid M., Aybek S. (2015). Interobserver agreement and validity of bedside ‘positive signs’ for functional weakness, sensory and gait disorders in conversion disorder: a pilot study. J. Neurol. Neurosurg. Psychiatry.

[b0100] Dewey B.E., Zhao C., Reinhold J.C., Carass A., Fitzgerald K.C., Sotirchos E.S., Saidha S., Oh J., Pham D.L., Calabresi P.A., van Zijl P.C.M., Prince J.L. (2019). DeepHarmony: a deep learning approach to contrast harmonization across scanner changes. Magn. Reson. Imaging.

[b0105] Di Martino A., Yan C.-G.-G., Li Q., Denio E., Castellanos F.X., Alaerts K., Anderson J.S., Assaf M., Bookheimer S.Y., Dapretto M., Deen B., Delmonte S., Dinstein I., Ertl-Wagner B., Fair D.A., Gallagher L., Kennedy D.P., Keown C.L., Keysers C., Lainhart J.E., Lord C., Luna B., Menon V., Minshew N.J., Monk C.S., Mueller S., Müller R.-A.-A., Nebel M.B., Nigg J.T., O’Hearn K., Pelphrey K.A., Peltier S.J., Rudie J.D., Sunaert S., Thioux M., Tyszka J.M., Uddin L.Q., Verhoeven J.S., Wenderoth N., Wiggins J.L., Mostofsky S.H., Milham M.P. (2014). The autism brain imaging data exchange: towards a large-scale evaluation of the intrinsic brain architecture in autism. Mol. Psychiatry.

[b0110] Diez I., Larson A.G., Nakhate V., Dunn E.C., Fricchione G.L., Nicholson T.R., Sepulcre J., Perez D.L. (2020). Early-life trauma endophenotypes and brain circuit–gene expression relationships in functional neurological (conversion) disorder. Mol. Psychiatry.

[b0115] Diez I., Ortiz-Terán L., Williams B., Jalilianhasanpour R., Ospina J.P., Dickerson B.C., Keshavan M.S., Lafrance W.C., Sepulcre J., Perez D.L. (2019). Corticolimbic fast-tracking: enhanced multimodal integration in functional neurological disorder. J. Neurol. Neurosurg. Psychiatry.

[b0120] Ding J.-R., An D., Liao W., Li J., Wu G.-R., Xu Q., Long Z., Gong Q., Zhou D., Sporns O., Chen H., Kaiser M. (2013). Altered functional and structural connectivity networks in psychogenic non-epileptic seizures. PLoS One.

[b0125] Donnelly-Kehoe P.A., Pascariello G.O., García A.M., Hodges J.R., Miller B., Rosen H., Manes F., Landin-Romero R., Matallana D., Serrano C., Herrera E., Reyes P., Santamaria-Garcia H., Kumfor F., Piguet O., Ibanez A., Sedeño L. (2019). Robust automated computational approach for classifying frontotemporal neurodegeneration: multimodal/multicenter neuroimaging. Alzheimer’s Dement. Diagnosis Assess. Dis. Monit..

[b0130] Drane D.L., Fani N., Hallett M., Khalsa S.S., Perez D.L., Roberts N.A. (2020). A framework for understanding the pathophysiology of functional neurological disorder. CNS Spectr..

[b0135] Dyrba M., Ewers M., Wegrzyn M., Kilimann I., Plant C., Oswald A., Meindl T., Pievani M., Bokde A.L.W., Fellgiebel A., Filippi M., Hampel H., Klöppel S., Hauenstein K., Kirste T., Teipel S.J., Zhan W. (2013). Robust automated detection of microstructural white matter degeneration in Alzheimer’s disease using machine learning classification of multicenter DTI data. PLoS One.

[b0140] Eickhoff S.B., Constable R.T., Yeo B.T.T. (2018). Topographic organization of the cerebral cortex and brain cartography. Neuroimage.

[b0145] Erickson B.J., Korfiatis P., Akkus Z., Kline T.L. (2017). Machine learning for medical imaging. RadioGraphics.

[b0150] Espay A.J., Aybek S., Carson A., Edwards M.J., Goldstein L.H., Hallett M., LaFaver K., LaFrance W.C., Lang A.E., Nicholson T., Nielsen G., Reuber M., Voon V., Stone J., Morgante F. (2018). Current concepts in diagnosis and treatment of functional neurological disorders. JAMA Neurol..

[b0155] Espay A.J., Goldenhar L.M., Voon V., Schrag A., Burton N., Lang A.E. (2009). Opinions and clinical practices related to diagnosing and managing patients with psychogenic movement disorders: an international survey of movement disorder society members. Mov. Disord..

[b0160] Espay A.J., Maloney T., Vannest J., Norris M.M., Eliassen J.C., Neefus E., Allendorfer J.B., Chen R., Szaflarski J.P. (2018). Dysfunction in emotion processing underlies functional (psychogenic) dystonia. Mov. Disord..

[b0165] Friedman L., Glover G.H., The FBIRN Consortium (2006). Reducing interscanner variability of activation in a multicenter fMRI study: Controlling for signal-to-fluctuation-noise-ratio (SFNR) differences. Neuroimage.

[b0170] Galli S., Béreau M., Magnin E., Moulin T., Aybek S. (2020). Functional movement disorders. Rev. Neurol. (Paris).

[b0175] Glasser M.F., Coalson T.S., Robinson E.C., Hacker C.D., Harwell J., Yacoub E., Ugurbil K., Andersson J., Beckmann C.F., Jenkinson M., Smith S.M., Van Essen D.C. (2016). A multi-modal parcellation of human cerebral cortex. Nature.

[b0180] Glover G.H., Mueller B.A., Turner J.A., Van Erp T.G.M., Liu T.T., Greve D.N., Voyvodic J.T., Rasmussen J., Brown G.G., Keator D.B., Calhoun V.D., Lee H.J., Ford J.M., Mathalon D.H., Diaz M., O’Leary D.S., Gadde S., Preda A., Lim K.O., Wible C.G., Stern H.S., Belger A., McCarthy G., Ozyurt B., Potkin S.G. (2012). Function biomedical informatics research network recommendations for prospective multicenter functional MRI studies. J. Magn. Reson. Imaging.

[b0185] Greicius M. (2008). Resting-state functional connectivity in neuropsychiatric disorders. Curr. Opin. Neurol..

[b0190] Gupta A., Lang A.E. (2009). Psychogenic movement disorders. Curr. Opin. Neurol..

[b0195] Guyon I., Elisseeff A., Kaelbling L.P. (2003). An introduction to variable and feature selection. J. Mach. Learn. Res..

[b0200] Hallett M., Aybek S., Dworetzky B.A., McWhirter L., Staab J.P., Stone J. (2022). Functional neurological disorder: new subtypes and shared mechanisms. Lancet Neurol..

[b0205] Hassa T., Sebastian A., Liepert J., Weiller C., Schmidt R., Tüscher O. (2017). Symptom-specific amygdala hyperactivity modulates motor control network in conversion disorder. NeuroImage Clin..

[b0210] Jack, C.R., Bernstein, M.A., Fox, N.C., Thompson, P., Alexander, G., Harvey, D., Borowski, B., Britson, P.J., Whitwell, J.L., Ward, C., Dale, A.M., Felmlee, J.P., Gunter, J.L., Hill, D.L.G., Killiany, R., Schuff, N., Fox-Bosetti, S., Lin, C., Studholme, C., DeCarli, C.S., Krueger, G., Ward, H.A., Metzger, G.J., Scott, K.T., Mallozzi, R., Blezek, D., Levy, J., Debbins, J.P., Fleisher, A.S., Albert, M., Green, R., Bartzokis, G., Glover, G., Mugler, J., Weiner, M.W., L. Whitwell, J., Ward, C., Dale, A.M., Felmlee, J.P., Gunter, J.L., Hill, D.L.G., Killiany, R., Schuff, N., Fox-Bosetti, S., Lin, C., Studholme, C., DeCarli, C.S., Gunnar Krueger, Ward, H.A., Metzger, G.J., Scott, K.T., Mallozzi, R., Blezek, D., Levy, J., Debbins, J.P., Fleisher, A.S., Albert, M., Green, R., Bartzokis, G., Glover, G., Mugler, J., Weiner, M.W., 2008. The Alzheimer’s disease neuroimaging initiative (ADNI): MRI methods. J. Magn. Reson. Imaging 27, 685–691. https://doi.org/10.1002/jmri.21049. 10.1002/jmri.21049 PMC2544629 18302232

[b0215] Longarzo M., Cavaliere C., Mele G., Tozza S., Tramontano L., Alfano V., Aiello M., Salvatore M., Grossi D. (2020). Microstructural changes in motor functional conversion disorder: multimodal imaging approach on a case. Brain Sci..

[b0220] Ludwig J., Speier P., Seifert F., Schaeffter T., Kolbitsch C. (2021). Pilot tone–based motion correction for prospective respiratory compensated cardiac cine MRI. Magn. Reson. Med..

[b0225] Ludwig L., Pasman J.A., Nicholson T., Aybek S., David A.S., Tuck S., Kanaan R.A., Roelofs K., Carson A., Stone J. (2018). Stressful life events and maltreatment in conversion (functional neurological) disorder: systematic review and meta-analysis of case-control studies. Lancet Psychiatry.

[b0230] Marapin R.S., Gelauff J.M., Marsman J.B.C., de Jong B.M., Dreissen Y.E.M., Koelman J.H.T.M., van der Horn H.J., Tijssen M.A.J. (2021). Altered posterior midline activity in patients with jerky and tremulous functional movement disorders. Brain Connect..

[b0235] Marapin R.S., van der Stouwe A.M.M., de Jong B.M., Gelauff J.M., Vergara V.M., Calhoun V.D., Dalenberg J.R., Dreissen Y.E.M., Koelman J.H.T.M., Tijssen M.A.J., van der Horn H.J. (2020). The chronnectome as a model for Charcot’s ‘dynamic lesion’ in functional movement disorders. NeuroImage Clin..

[b0240] Maurer C.W., LaFaver K., Ameli R., Epstein S.A., Hallett M., Horovitz S.G. (2016). Impaired self-agency in functional movement disorders: a resting-state fMRI study. Neurology.

[b0245] Monsa R., Peer M., Arzy S. (2018). Self-reference, emotion inhibition and somatosensory disturbance: preliminary investigation of network perturbations in conversion disorder. Eur. J. Neurol..

[b0250] Mori Y., Miyata J., Isobe M., Son S., Yoshihara Y., Aso T., Kouchiyama T., Murai T., Takahashi H. (2018). Effect of phase-encoding direction on group analysis of resting-state functional magnetic resonance imaging. Psychiatry Clin. Neurosci..

[b0255] Morris L.S., To B., Baek K., Chang-Webb Y.-C., Mitchell S., Strelchuk D., Mikheenko Y., Phillips W., Zandi M., Jenaway A., Walsh C., Voon V. (2017). Disrupted avoidance learning in functional neurological disorder: Implications for harm avoidance theories. NeuroImage Clin..

[b0260] Mueller K., Růžička F., Slovák M., Forejtová Z., Dušek P., Dušek P., Jech R., Serranová T. (2022). Symptom-severity-related brain connectivity alterations in functional movement disorders. NeuroImage Clin..

[b0265] Nakano T., Takamura M., Ichikawa N., Okada G., Okamoto Y., Yamada M., Suhara T., Yamawaki S., Yoshimoto J. (2020). Enhancing multi-center generalization of machine learning-based depression diagnosis from resting-state fMRI. Front. Psychiatry.

[b0270] Nielsen A.N., Barch D.M., Petersen S.E., Schlaggar B.L., Greene D.J. (2020). Machine learning with neuroimaging: evaluating its applications in psychiatry. Biol. Psychiatry Cogn. Neurosci. Neuroimaging.

[b0275] Nielsen G., Ricciardi L., Meppelink A.M., Holt K., Teodoro T., Edwards M. (2017). A simplified version of the psychogenic movement disorders rating scale: the simplified functional movement disorders rating scale (S-FMDRS). Mov. Disord. Clin. Pract..

[b0280] Noble S., Scheinost D., Finn E.S., Shen X., Papademetris X., McEwen S.C., Bearden C.E., Addington J., Goodyear B., Cadenhead K.S., Mirzakhanian H., Cornblatt B.A., Olvet D.M., Mathalon D.H., McGlashan T.H., Perkins D.O., Belger A., Seidman L.J., Thermenos H., Tsuang M.T., van Erp T.G.M., Walker E.F., Hamann S., Woods S.W., Cannon T.D., Constable R.T. (2017). Multisite reliability of MR-based functional connectivity. Neuroimage.

[b0285] Nunes A., Schnack H.G., Ching C.R.K., Agartz I., Akudjedu T.N., Alda M., Alnæs D., Alonso-Lana S., Bauer J., Baune B.T., Bøen E., Bonnin C.D.M., Busatto G.F., Canales-Rodríguez E.J., Cannon D.M., Caseras X., Chaim-Avancini T.M., Dannlowski U., Díaz-Zuluaga A.M., Dietsche B., Doan N.T., Duchesnay E., Elvsåshagen T., Emden D., Eyler L.T., Fatjó-Vilas M., Favre P., Foley S.F., Fullerton J.M., Glahn D.C., Goikolea J.M., Grotegerd D., Hahn T., Henry C., Hibar D.P., Houenou J., Howells F.M., Jahanshad N., Kaufmann T., Kenney J., Kircher T.T.J., Krug A., Lagerberg T.V., Lenroot R.K., López-Jaramillo C., Machado-Vieira R., Malt U.F., McDonald C., Mitchell P.B., Mwangi B., Nabulsi L., Opel N., Overs B.J., Pineda-Zapata J.A., Pomarol-Clotet E., Redlich R., Roberts G., Rosa P.G., Salvador R., Satterthwaite T.D., Soares J.C., Stein D.J., Temmingh H.S., Trappenberg T., Uhlmann A., van Haren N.E.M., Vieta E., Westlye L.T., Wolf D.H., Yüksel D., Zanetti M.V., Andreassen O.A., Thompson P.M., Hajek T. (2020). Using structural MRI to identify bipolar disorders – 13 site machine learning study in 3020 individuals from the ENIGMA Bipolar Disorders Working Group. Mol. Psychiatry.

[b0290] Orrù G., Pettersson-Yeo W., Marquand A.F., Sartori G., Mechelli A. (2012). Using Support Vector Machine to identify imaging biomarkers of neurological and psychiatric disease: a critical review. Neurosci. Biobehav. Rev..

[b0295] Patel M.J., Andreescu C., Price J.C., Edelman K.L., Reynolds C.F., Aizenstein H.J. (2015). Machine learning approaches for integrating clinical and imaging features in late-life depression classification and response prediction. Int. J. Geriatr. Psychiatry.

[b0300] Perez D.L., Barsky A.J., Daffner K., Silbersweig D.A. (2012). Motor and somatosensory conversion disorder: a functional unawareness Syndrome?. J. Neuropsychiatry Clin. Neurosci..

[b0305] Perez, D.L., Matin, N., Barsky, A., Costumero-ramos, V., Makaretz, S.J., Young, S.S., Sepulcre, J., LaFrance, W.C., Keshavan, M.S., Dickerson, B.C., LaFranceJr, W.C., Keshavan, M.S., Dickerson, B.C., Sara, J., Young, S.S., Sepulcre, J., Jr, W.C.L., Matcheri, S., Dickerson, B.C., 2017. Cingulo-insular structural alterations associated with psychogenic symptoms, childhood abuse and PTSD in functional neurological disorders. J. Neurol. Neurosurg. Psychiatry 88, 491–497. https://doi.org/10.1136/jnnp-2016-314998. 10.1136/jnnp-2016-314998 PMC5497745 28416565

[b0310] Pessoa L. (2008). On the relationship between emotion and cognition. Nat. Rev. Neurosci..

[b0315] Power J.D., Mitra A., Laumann T.O., Snyder A.Z., Schlaggar B.L., Petersen S.E. (2014). Methods to detect, characterize, and remove motion artifact in resting state fMRI. Neuroimage.

[b0320] Richiardi J., Eryilmaz H., Schwartz S., Vuilleumier P., Van De Ville D. (2011). Decoding brain states from fMRI connectivity graphs. Neuroimage.

[b0325] Richiardi J., Van De Ville D., Riesen K., Bunke H. (2010). Vector space embedding of undirected graphs with fixed-cardinality vertex sequences for classification. Proc. - Int. Conf. Pattern Recognit..

[b0330] Rozycki M., Satterthwaite T.D., Koutsouleris N., Erus G., Doshi J., Wolf D.H., Fan Y., Gur R.E., Gur R.C., Meisenzahl E.M., Zhuo C., Yin H., Yan H., Yue W., Zhang D., Davatzikos C. (2018). Multisite machine learning analysis provides a robust structural imaging signature of schizophrenia detectable across diverse patient populations and within individuals. Schizophr. Bull..

[b0335] Smith S.M., Miller K.L., Salimi-Khorshidi G., Webster M., Beckmann C.F., Nichols T.E., Ramsey J.D., Woolrich M.W. (2011). Network modelling methods for FMRI. Neuroimage.

[b0340] Sokolov, A.A., Granziera, C., Fischi-Gomez, E., Preti, M.G., Ryvlin, P., Van De Ville, D., Friston, K.J., 2019. Brain network analyses in clinical neuroscience. Swiss Arch. Neurol. Psychiatry Psychother. https://doi.org/10.4414/sanp.2019.03074.

[b0345] Spielberger, C., Gorsuch, R., Lushene, R., Vagg, P., Jacobs, G., 1983. Manual for the State-Trait Anxiety Inventory (Form Y1 - Y2).

[b0350] Stone J., Carson A. (2015). Functional neurologic disorders. Contin. Lifelong Learn. Neurol..

[b0355] Stone J., LaFrance W.C., Brown R., Spiegel D., Levenson J.L., Sharpe M. (2011). Conversion disorder: current problems and potential solutions for DSM-5. J. Psychosom. Res..

[b0360] Stone J., Zeman A., Simonotto E., Meyer M., Azuma R., Flett S., Sharpe M. (2007). fMRI in patients with motor conversion symptoms and controls with simulated weakness. Psychosom. Med..

[b0365] Syed T.U., LaFrance W.C., Kahriman E.S., Hasan S.N., Rajasekaran V., Gulati D., Borad S., Shahid A., Fernandez-Baca G., Garcia N., Pawlowski M., Loddenkemper T., Amina S., Koubeissi M.Z. (2011). Can semiology predict psychogenic nonepileptic seizures? A prospective study. Ann. Neurol..

[b0370] Szaflarski J.P., Allendorfer J.B., Nenert R., LaFrance W.C., Barkan H.I., DeWolfe J., Pati S., Thomas A.E., Ver Hoef L. (2018). Facial emotion processing in patients with seizure disorders. Epilepsy Behav..

[b0375] Takamura T., Hanakawa T. (2017). Clinical utility of resting-state functional connectivity magnetic resonance imaging for mood and cognitive disorders. J. Neural Transm..

[b0380] Teipel S.J., Wohlert A., Metzger C., Grimmer T., Sorg C., Ewers M., Meisenzahl E., Klöppel S., Borchardt V., Grothe M.J., Walter M., Dyrba M. (2017). Multicenter stability of resting state fMRI in the detection of Alzheimer’s disease and amnestic MCI. NeuroImage Clin..

[b0385] Tzourio-Mazoyer N., Landeau B., Papathanassiou D., Crivello F., Etard O., Delcroix N., Mazoyer B., Joliot M. (2002). Automated anatomical labeling of activations in SPM using a macroscopic anatomical parcellation of the MNI MRI single-subject brain. Neuroimage.

[b0390] Vabalas A., Gowen E., Poliakoff E., Casson A.J., Hernandez-Lemus E. (2019). Machine learning algorithm validation with a limited sample size. PLoS One.

[b0395] van den Heuvel M.P., Hulshoff Pol H.E. (2010). Exploring the brain network: a review on resting-state fMRI functional connectivity. Eur. Neuropsychopharmacol..

[b0400] van der Kruijs S.J.M., Bodde N.M.G., Vaessen M.J., Lazeron R.H.C., Vonck K., Boon P., Hofman P.A.M., Backes W.H., Aldenkamp A.P., Jansen J.F.A. (2012). Functional connectivity of dissociation in patients with psychogenic non-epileptic seizures. J. Neurol. Neurosurg. Psychiatry.

[b0405] Van Dijk K.R.A., Sabuncu M.R., Buckner R.L. (2012). The influence of head motion on intrinsic functional connectivity MRI. Neuroimage.

[b0410] Vasta R., Cerasa A., Sarica A., Bartolini E., Martino I., Mari F., Metitieri T., Quattrone A., Gambardella A., Guerrini R., Labate A. (2018). The application of artificial intelligence to understand the pathophysiological basis of psychogenic nonepileptic seizures. Epilepsy Behav..

[b0415] Voon V., Brezing C., Gallea C., Hallett M. (2011). Aberrant supplementary motor complex and limbic activity during motor preparation in motor conversion disorder. Mov. Disord..

[b0420] Voon V., Cavanna A.E., Coburn K., Sampson S., Reeve A., LaFrance W.C. (2016). Functional neuroanatomy and neurophysiology of functional neurological disorders (Conversion disorder). J. Neuropsychiatry Clin. Neurosci..

[b0425] Voon V., Gallea C., Hattori N., Bruno M., Ekanayake V., Hallett M. (2010). The involuntary nature of conversion disorder. Neurology.

[b0430] Wegrzyk J., Kebets V., Richiardi J., Galli S., de Ville D., Van A., S., (2018). Identifying motor functional neurological disorder using resting-state functional connectivity. NeuroImage Clin..

[b0435] World Health Organization (1993).

[b0440] Xia M., Wang J., He Y., Csermely P. (2013). BrainNet viewer: a network visualization tool for human brain connectomics. PLoS One.

[b0445] Yamashita A., Yahata N., Itahashi T., Lisi G., Yamada T., Ichikawa N., Takamura M., Yoshihara Y., Kunimatsu A., Okada N., Yamagata H., Matsuo K., Hashimoto R., Okada G.o., Sakai Y., Morimoto J., Narumoto J., Shimada Y., Kasai K., Kato N., Takahashi H., Okamoto Y., Tanaka S.C., Kawato M., Yamashita O., Imamizu H., Macleod M.R. (2019). Harmonization of resting-state functional MRI data across multiple imaging sites via the separation of site differences into sampling bias and measurement bias. PLoS Biol..

[b0450] Yu M., Linn K.A., Cook P.A., Phillips M.L., McInnis M., Fava M., Trivedi M.H., Weissman M.M., Shinohara R.T., Sheline Y.I. (2018). Statistical harmonization corrects site effects in functional connectivity measurements from multi-site fMRI data. Hum. Brain Mapp..

[b0455] Zeng L.-L.-L., Wang H., Hu P., Yang B., Pu W., Shen H., Chen X., Liu Z., Yin H., Tan Q., Wang K., Hu D. (2018). Multi-site diagnostic classification of schizophrenia using discriminant deep learning with functional connectivity MRI. EBioMedicine.

[b0460] Zhao N., Yuan L.-X.-X., Jia X.-Z.-Z., Zhou X.-F.-F., Deng X.-P.-P., He H.-J.-J., Zhong J., Wang J., Zang Y.-F.-F. (2018). Intra- and inter-scanner reliability of voxel-wise whole-brain analytic metrics for resting state fMRI. Front. Neuroinform..

[b0465] Zhuang H., Liu R., Wu C., Meng Z., Wang D., Liu D., Liu M., Li Y. (2019). Multimodal classification of drug-naïve first-episode schizophrenia combining anatomical, diffusion and resting state functional resonance imaging. Neurosci. Lett..

