# Supplementary material for: Multi-centre classification of functional neurological disorders based on resting-state functional connectivity
Source: Neuroimage Clin. 2022 Jun 17;35:103090. doi: 10.1016/j.nicl.2022.103090 (PMC9240866; doi:10.1016/j.nicl.2022.103090)
Supplement: Supplementary data 1 [file mmc1.docx]

**Supplementary Material**

**Appendix 1:**

*Conversion to CGI score:* The CGI score of centre II and IV as well as the S-FMDRS score of centre III was converted into the same CGI score as in centre I.

| CGI (centre I) |  | CGI (centre II, IV) | S-FMDRS (centre III) |
| --- | --- | --- | --- |
| 0 = none | 0 | = none | 0 = none |
|  | 1 |  |  |
| 1 = mild | 2 | = mild | 1 - 9 = mild |
|  | 3 |  |  |
| 2 = moderate | 4 | = moderate | 10 - 18 = moderate |
| 3 = severe | 5 | = severe | 19- 36 = severe |
|  | 6 |  |  |
| 4 = very severe | 7 | = very severe | 37 - 54 = very severe |

**Appendix 2:**

*Optimal Filter*

Each centre was pre-processed individually. Based on previous work of Richiardi and colleagues (Richiardi et al., 2011), we calculated a functional atlas based on a structural atlas. The structural atlas only served as a basis to compute the subject-specific low-resolution functional atlas. We extracted the region-averaged time-course from the voxels which correspond to the individual regions. Assuming *t* timepoints, we have then a tensor matrix for each centre of size

$X:t\times r\times s$, (1)

with *r* equals the number of regions and *s* equals the number of subjects. For the **intra-centre cross-validation** setting, we decided to explore the different filter options because of the potential differences in the functional connectivity graphs in different frequency subbands. Such differences might arise from the scanners itself such as mechanical resonance, manufacturer and model of the scanner, scanner sequence, etc.). Doing so, we can explore the diversity in classification performance within different filters and subbands, in which the classifier might build a model with substantially different parameters. Therefore, we optimized each centre independently.

To do so, we used two different filtering pipelines **Fig. 1**. In the first pipeline, the data was filtered in the time domain using a bandpass filter 0.01-0.08 Hz, which is most commonly used for resting-state data. In a second pipeline, the same data was filtered using a discrete wavelet transform along the temporal dimension, as it has been done in the previous project (Wegrzyk et al., 2018). Five frequency subbands were extracted with main bandpass characteristics at 0.125–0.25 Hz, 0.0625–0.125 Hz, 0.0312–0.0625 Hz, 0.0156 – 0.0312 Hz, and 0.0078 – 0.0156 Hz based on the repetition time (TR) = 2000ms.

During the **pooled- and inter-scanner cross-validation**, however, we used the bandpass-filtered data, in order to maintain a uniform pre-processing pipeline across all the four centres.

Upon filtering, we then used the filtered region-averaged time courses to compute functional connectivity (i.e., Pearson correlation) between different regions of interests (ROIs) leading to an $r\times r$ matrix for each subject. We then used direct graph embedding, as described in (Richiardi et al., 2011), in order to build our feature vectors.

*Optimal Filter Selection for Classification*

We explored the performance on the classification for the different filters and subbands of filters. The best performance in the intra-centre cross-validation was achieved using bandpass filter for centre I, wavelets filter subband 2 for centre II, and wavelets subband 5 for centre III and IV. These results are presented in the paper.

**
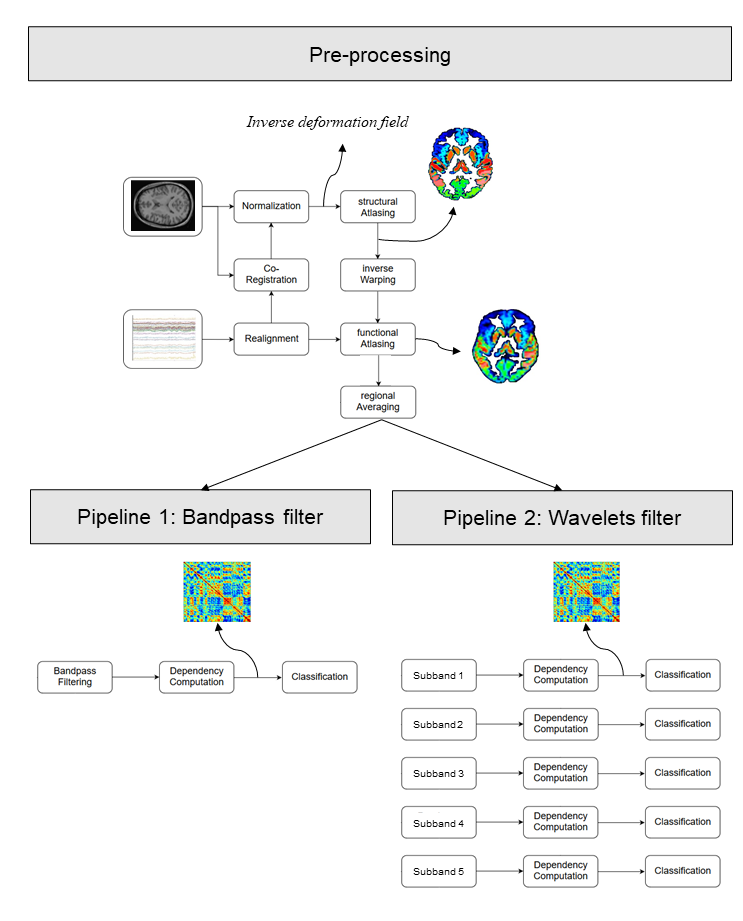
**

**Fig. 1.** Workflow of filtering pipelines. For the intra-centre cross-validation settings both pipelines were applied to the data and different classification performances (based on different filtering pipelines) were examined. For pooled- and inter-centre cross-validation, only pipeline 1 (bandpass filter) was used in order to maintain a uniform pre-processing pipeline across all centres.

**Supplemental Tables and Figures**

**Figure S1.** Mean Framewise displacement (FD) per centre with ***p ≤ 0.001, ** p ≤ 0.01, * p ≤ 0.05. FD measures showed a significant main effect of *centre* (F(3,164) = 5.5, p = 0.001). Post-hoc multiple comparison of means showed that the difference between centre I and centre III (p < 0.0001) and centre IV (p = 0.0006), as well as between centre II and centre III (p = 0.0002) and IV (p = 0.008) were statistically significant (Fig. 3).


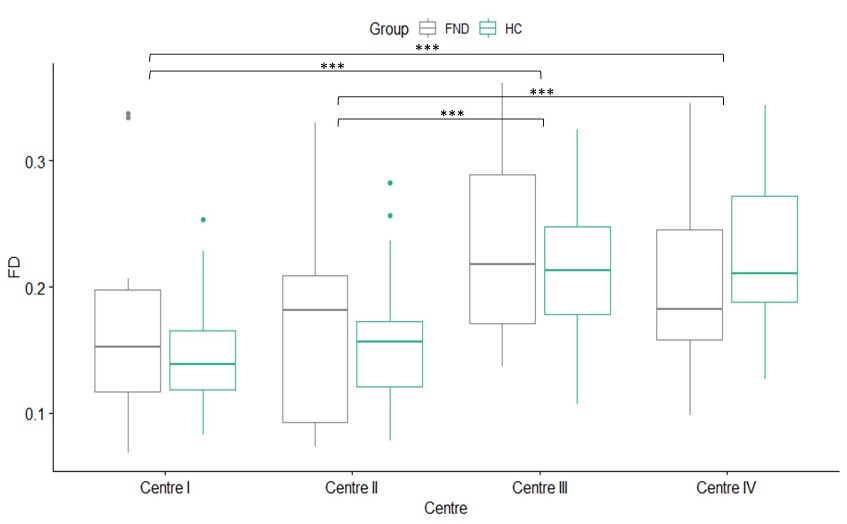


**Figure S2.** (A) Accuracy, (B) Sensitivity, and (C) Specificity with 95% confidence interval (95% - CI) of the different classification settings.

**(A) Accuracy**

**Setting 95% - CI**


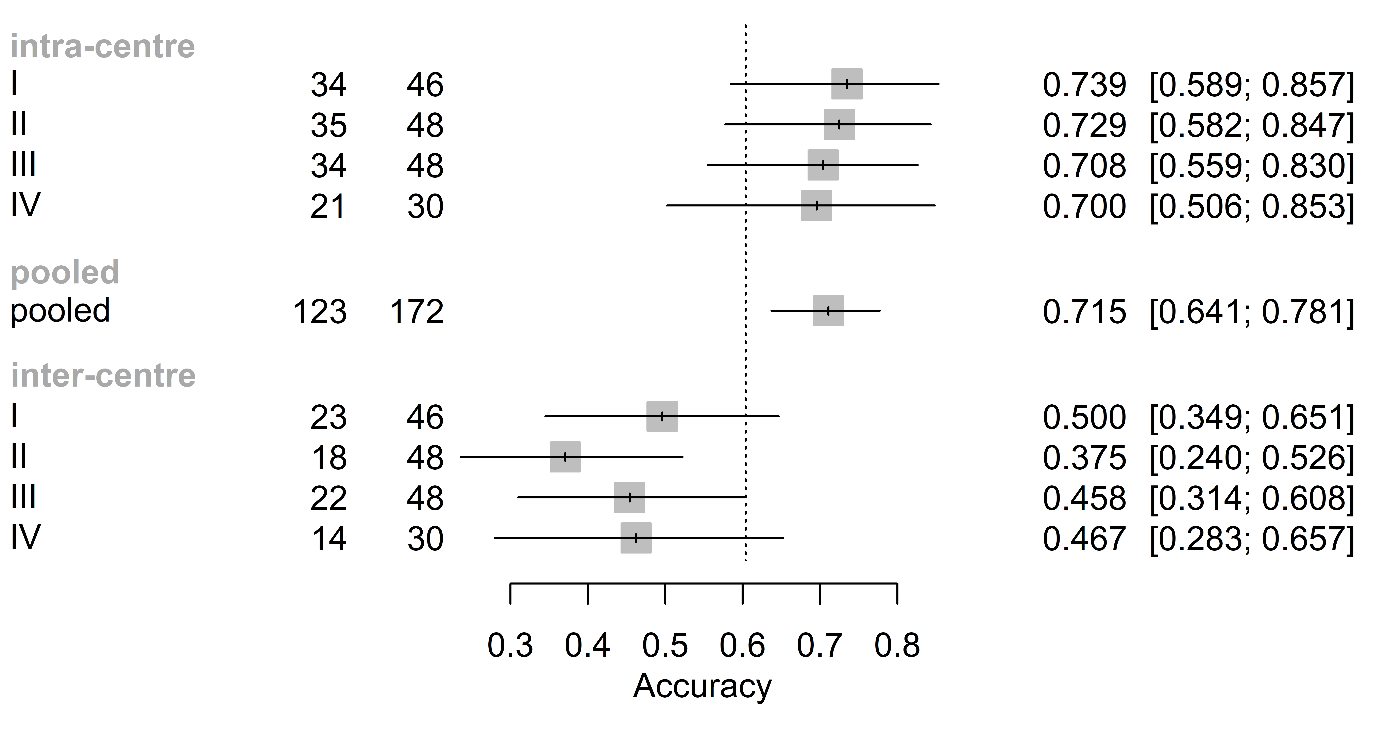


**(B) Sensitivity**

**Setting** TP TP+FN  **95% - CI**


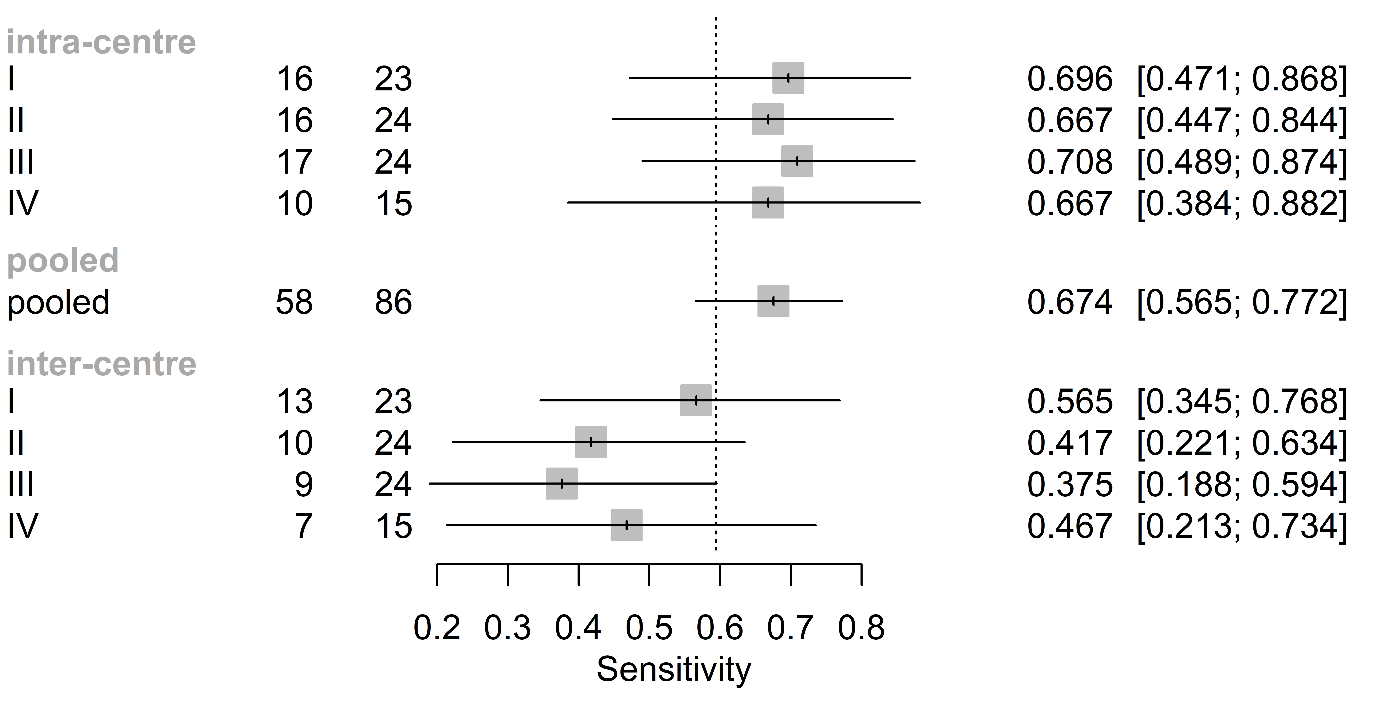


**(C) Specificity**

**Setting** TN TN+FP  **95% - CI**


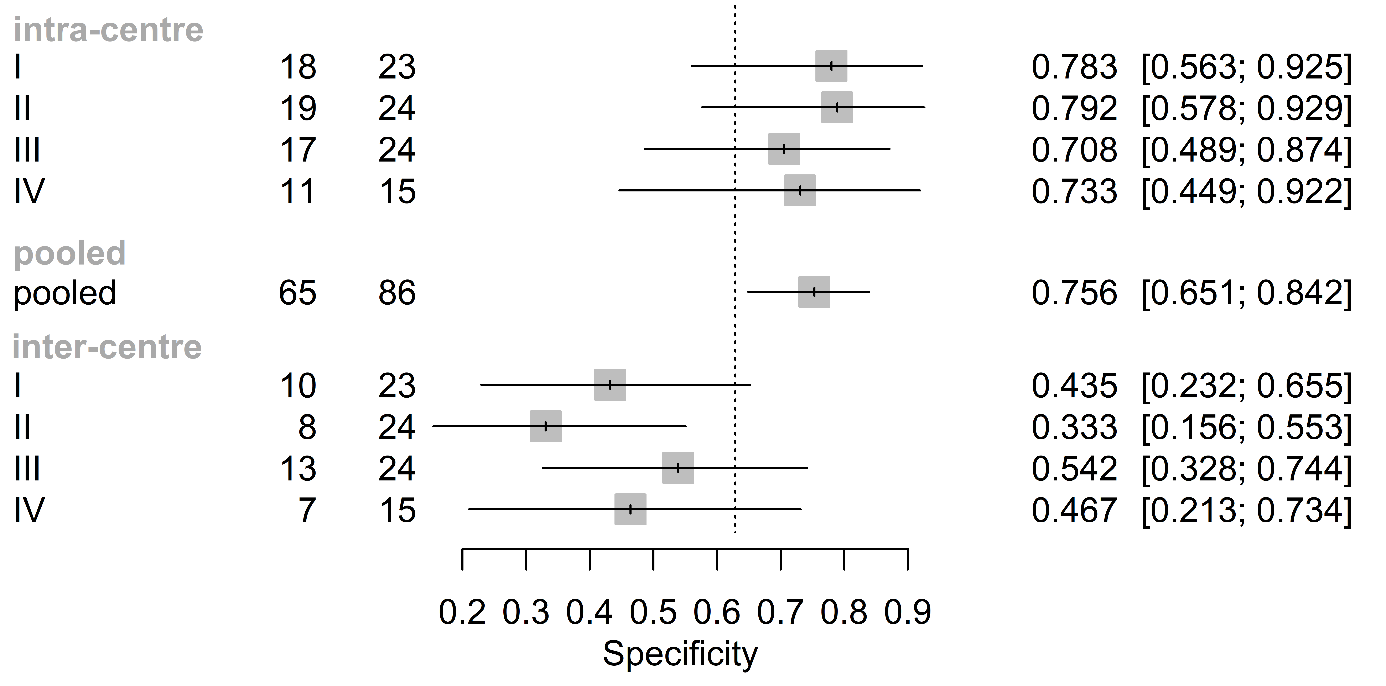


**Figure S3.** Area-under-the-curve (AUC) with 95% confidence interval (95% - CI) for the individual classification obtained with each setting. (A) Intra-centre cross-validation classification, and (B) inter-centre cross-validation classification.


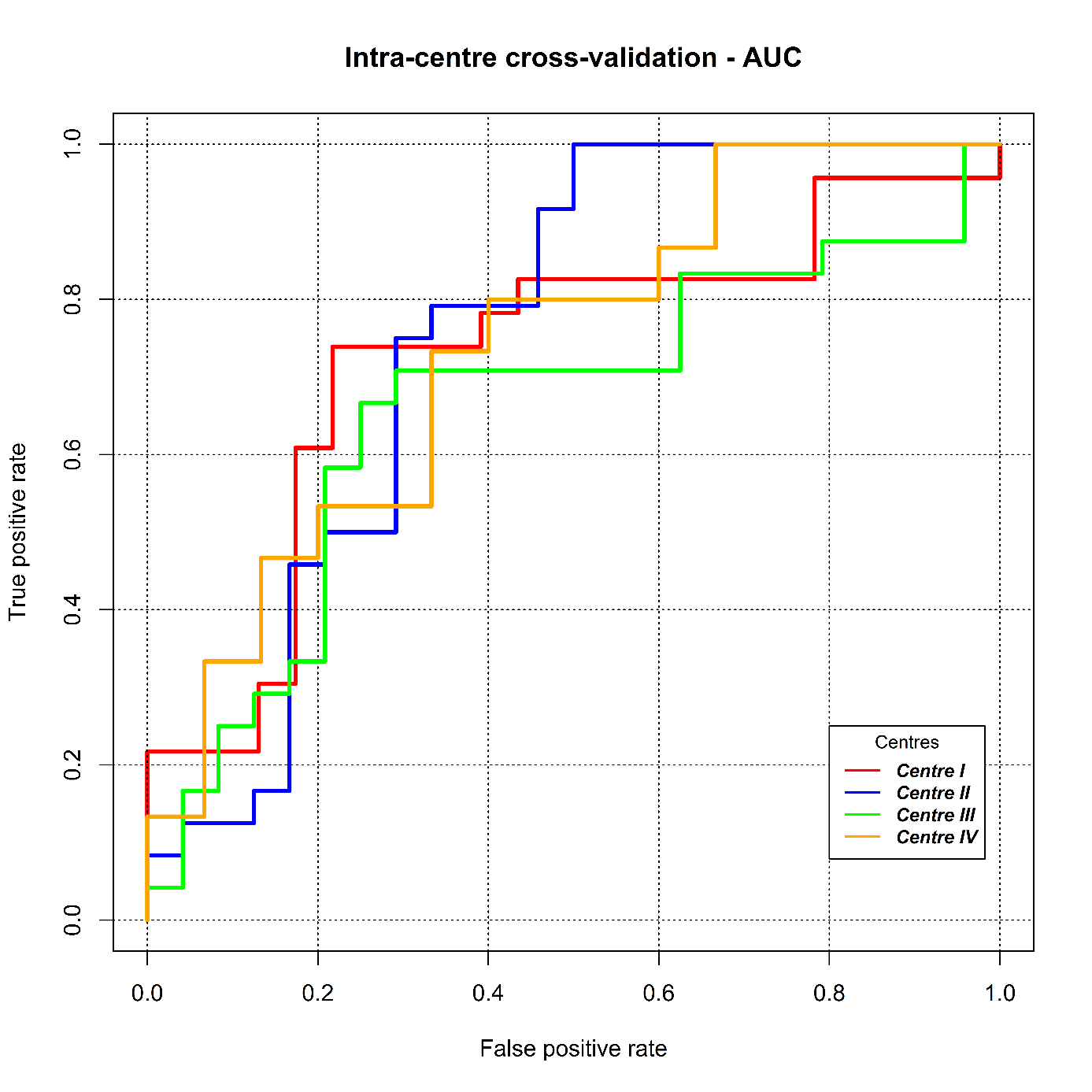


**(A)**


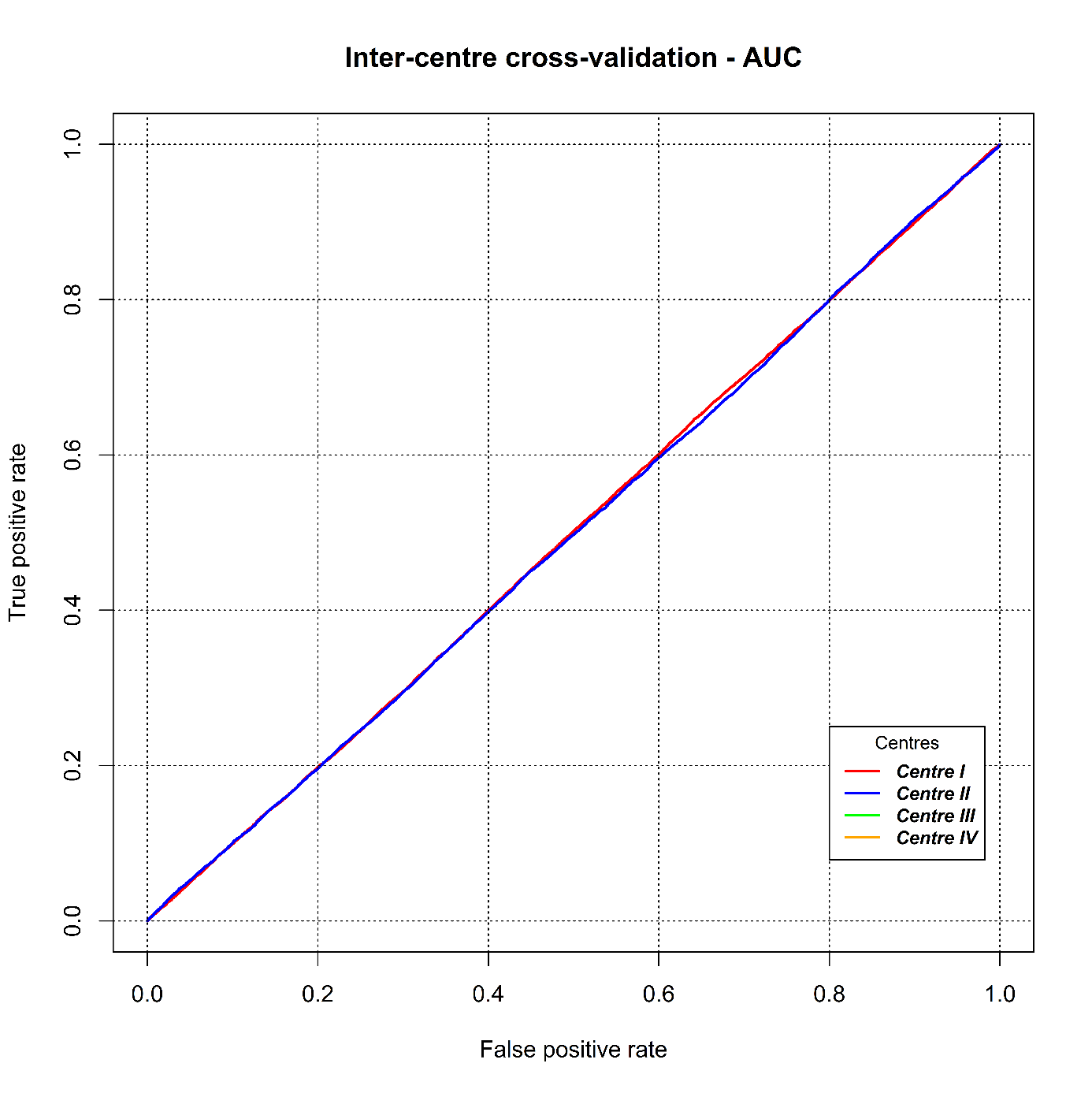


**(B)**

**Figure S4.** Functional connectivity of regions yielding the most discriminative connections of the pooled classification based on the AAL atlas. Size of the nodes correspond to nodal degree, respectively occurrence within the most discriminative connections. Colour of the nodes corresponds to different lobes of the AAL. Color of the edges correspond to functional connectivity between the regions, i.e., red displaying hyperconnected and blue hypoconnected in patients compared to controls.


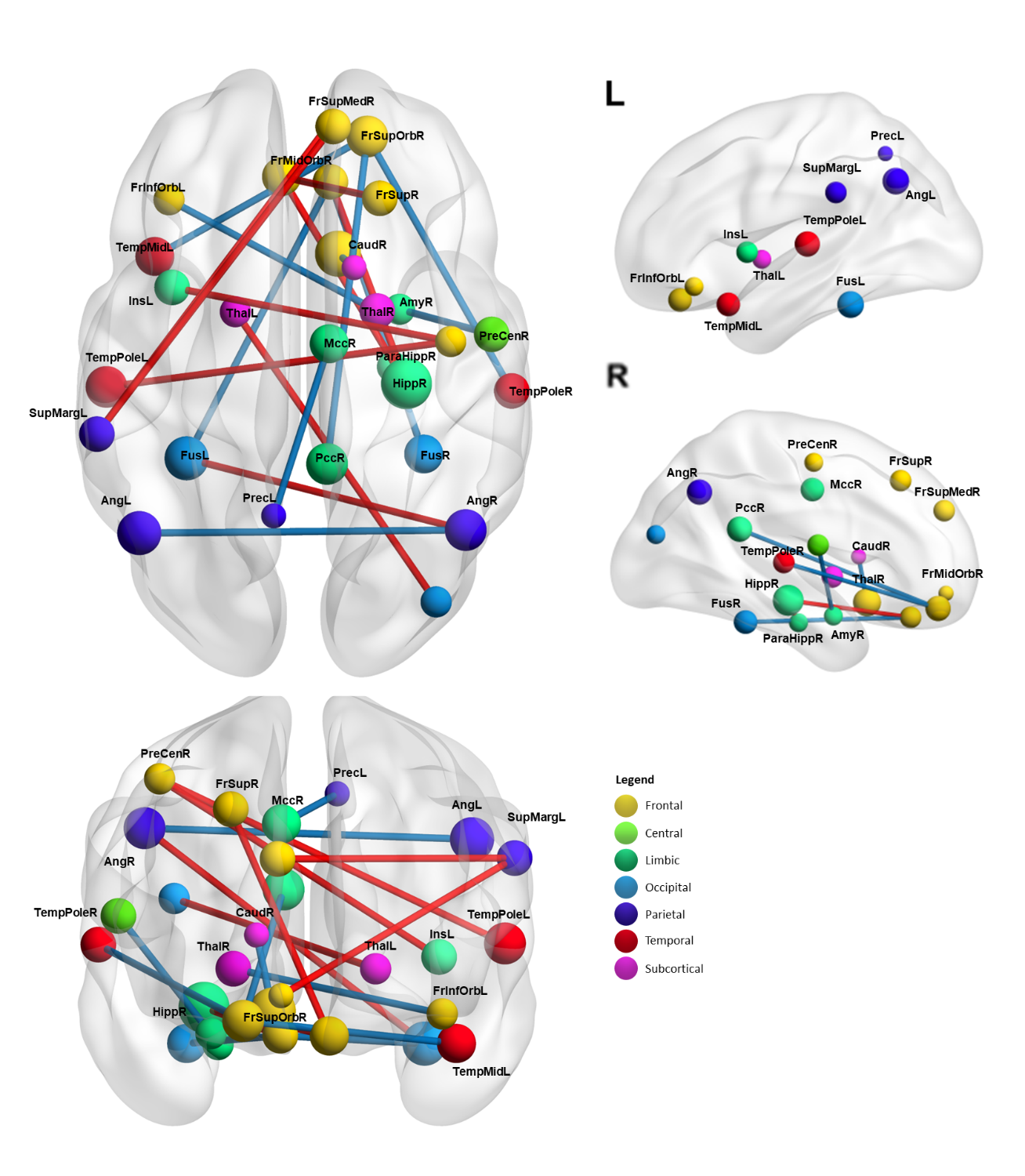


**Table S1.** Mean functional connectivity in controls and patients between pairs of regions showing discriminative functional connectivity.

|  |  | **HC** | **FND** |
| --- | --- | --- | --- |
| Hippocampus_R | Rectus_L | 0.182 | -0.047 |
|  | Parahippocampal_R | 0.352 | 0.115 |
|  | Rectus_R | 0.187 | -0.069 |
|  | Temporal_Pole_Sup_R | 0.218 | 0.048 |
|  | Parahippocampal_L | 0.287 | 0.085 |
|  | Frontal_Inf_Orb_R | 0.127 | -0.028 |
|  | Amygdala_L | 0.236 | 0.112 |
|  | Putamen_L | 0.084 | -0.042 |
|  | Temporal_Sup_R | 0.095 | 0.401 |
| Angular_L | Angular_R | 0.485 | 0.260 |
|  | Occipital_Sup_L | -0.298 | 0.102 |
|  | Frontal_Sup_R | 0.322 | -0.069 |
|  | Lingual_L | -0.270 | 0.111 |
|  | Fusiform_L | -0.210 | 0.124 |
|  | Frontal_Mid_Orb_Medial_L | 0.296 | 0.084 |
| Angular_R | Fusiform_L | -0.184 | 0.139 |
|  | Parietal_Inf_L | 0.052 | 0.269 |
|  | Precuneus_R | 0.165 | 0.413 |
|  | Fusiform_R | -0.188 | 0.099 |
|  | Postcentral_L | -0.0207 | 0.128 |
| Cingulum_Mid_R | Precuneus_L | 0.018 | 0.308 |
|  | Frontal_Sup_R | 0.065 | 0.279 |
|  | Frontal_Mid_Orb_Medial_L | -0.026 | 0.020 |
| Cingulum_Post_R | Frontal_Sup_Orb_R | -0.126 | 0.116 |
|  | Occipital_Sup_R | -0.187 | 0.103 |
|  | Insula_R | -0.096 | -0.139 |
|  | Thalamus_R | -0.002 | 0.182 |
|  | Frontal_Sup_Orb_L | -0.063 | 0.055 |
|  | Frontal_Inf_Tri_R | -0.102 | -0.056 |
| Cingulum_Ant_R | Caudate_R | 0.220 | 0.023 |
| Amygdala_R | Frontal_Mid_L | -0.061 | -0.019 |
|  | Temporal_Inf_R | -0.054 | 0.184 |
|  | Amygdala_L | 0.346 | 0.108 |
| Hippocampus_L | Cingulum_Mid_L | -0.015 | 0.081 |
|  | Temporal_Pole_Mid_L | 0.068 | 0.140 |
|  | Precuneus_R | -0.009 | 0.135 |
|  | Cingulum_Post_L | 0.009 | 0.213 |
|  | Precuneus_L | 0.012 | 0.173 |
|  | Amygdala_R | 0.203 | 0.105 |
|  | Parahippocampal_R | 0.296 | 0.122 |
|  | Temporal_Pole_Mid_R | 0.037 | 0.168 |
| SupraMarginal_R | Frontal_Mid_Orb_R | -0.037 | 0.101 |
|  | Temporal_Mid_L | -0.133 | 0.064 |
|  | Putamen_R | 0.009 | 0.216 |
| SupraMarginal_L | Frontal_Mid_L | 0.059 | 0.053 |
|  | Frontal_Inf_Oper_L | 0.267 | 0.171 |
| Fusiform_L | Frontal_Mid_Orb_Medial_L | -0.188 | 0.191 |
|  | Frontal_Mid_Orb_R | -0.178 | 0.308 |
|  | Thalamus_R | -0.032 | 0.119 |
|  | Cingulum_Post_R | -0.120 | 0.117 |
|  | Frontal_Mid_Orb_L | -0.179 | 0.270 |


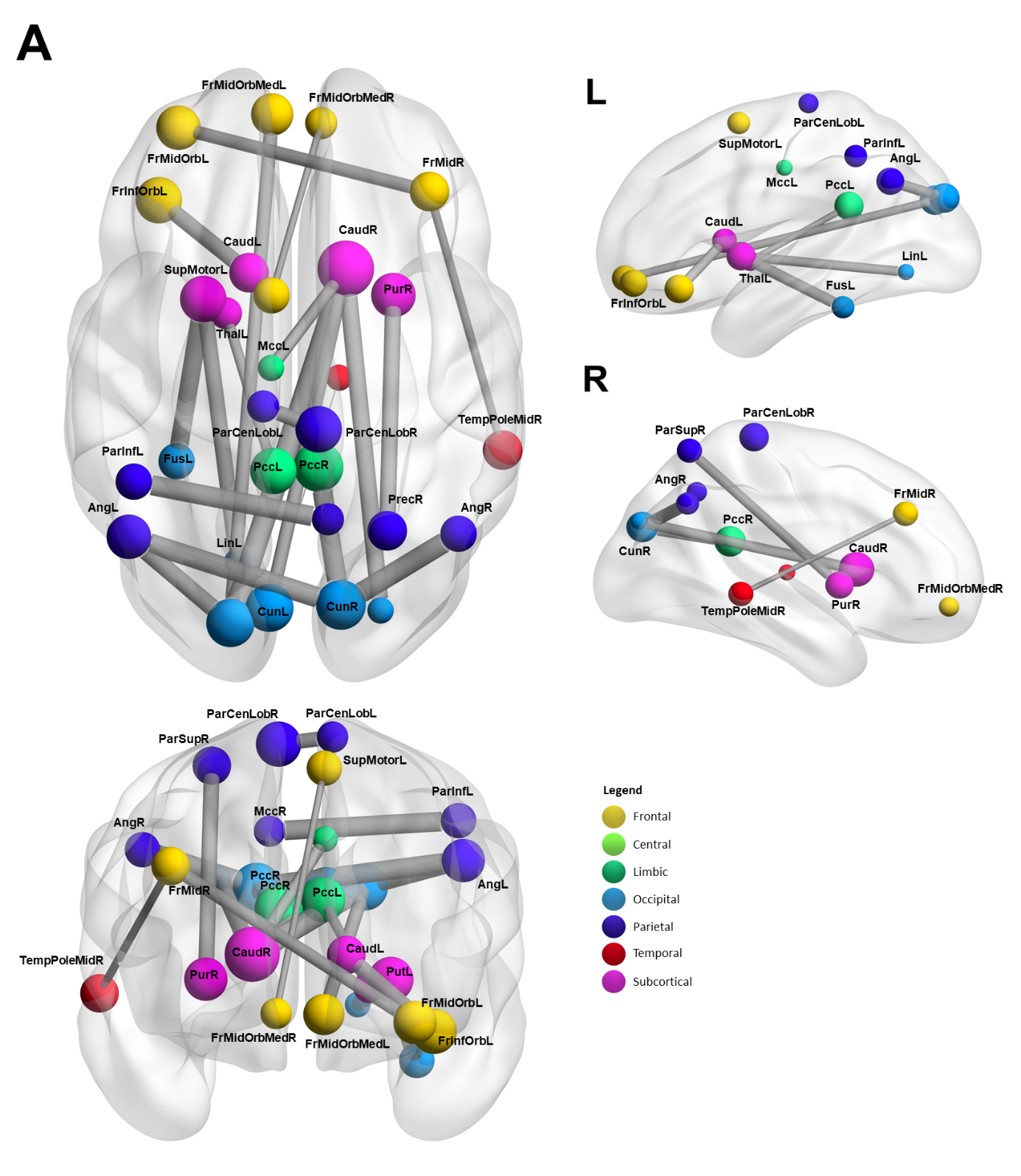
**Figure S5.** Regions yielding the most discriminative connections for each centre based on the AAL atlas. Size of the nodes correspond to nodal degree, respectively occurrence within the 200 most discriminative connections. Colour of the nodes corresponds to different lobes of the AAL. Thickness of edges correspond to SVM weights. Thicker edges therefore indicate higher SVM weights, respectively higher discrimination power. **A)** Centre I, **B)** Centre II, **C)** Centre III, and **D)** Centre IV.

**
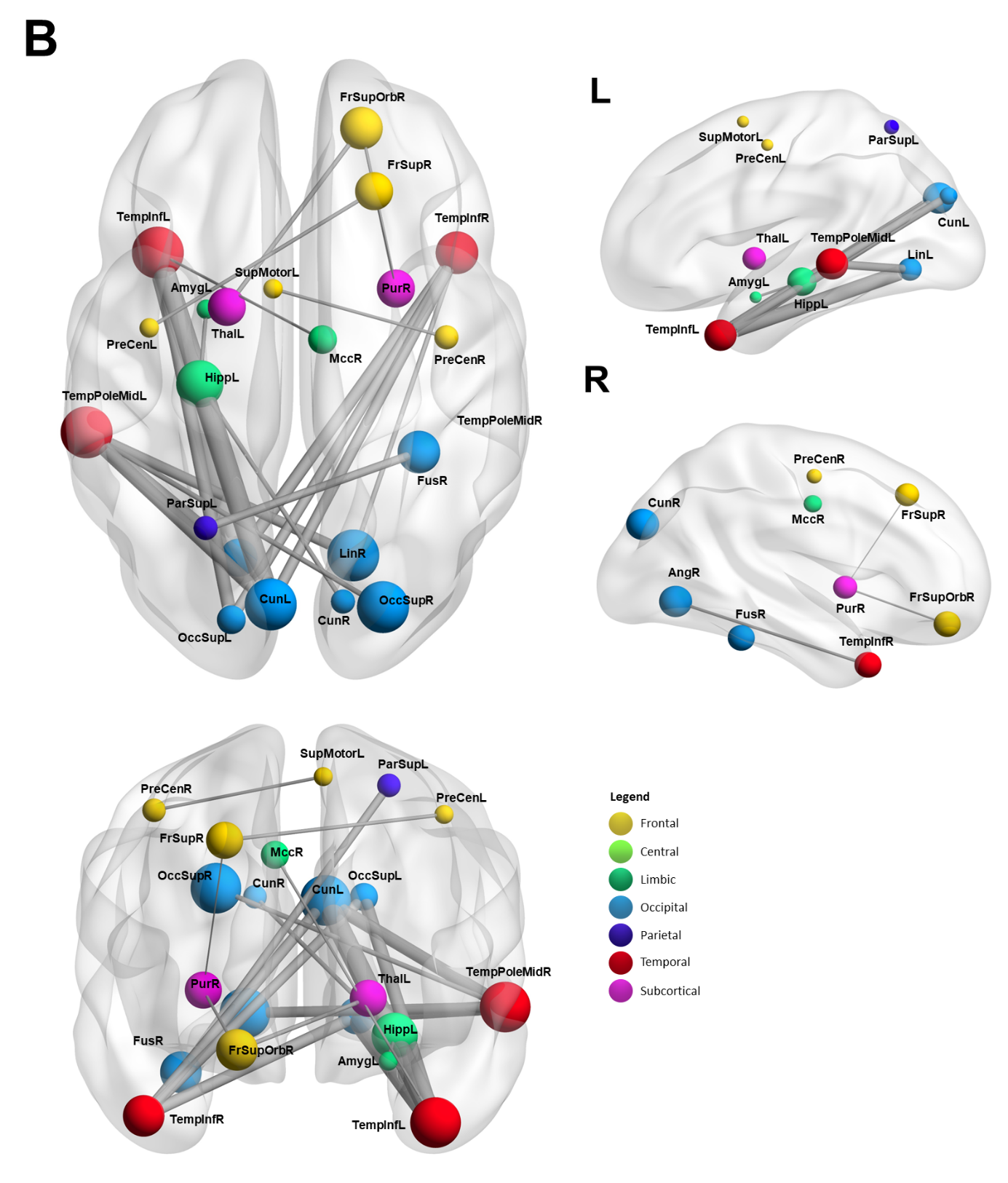

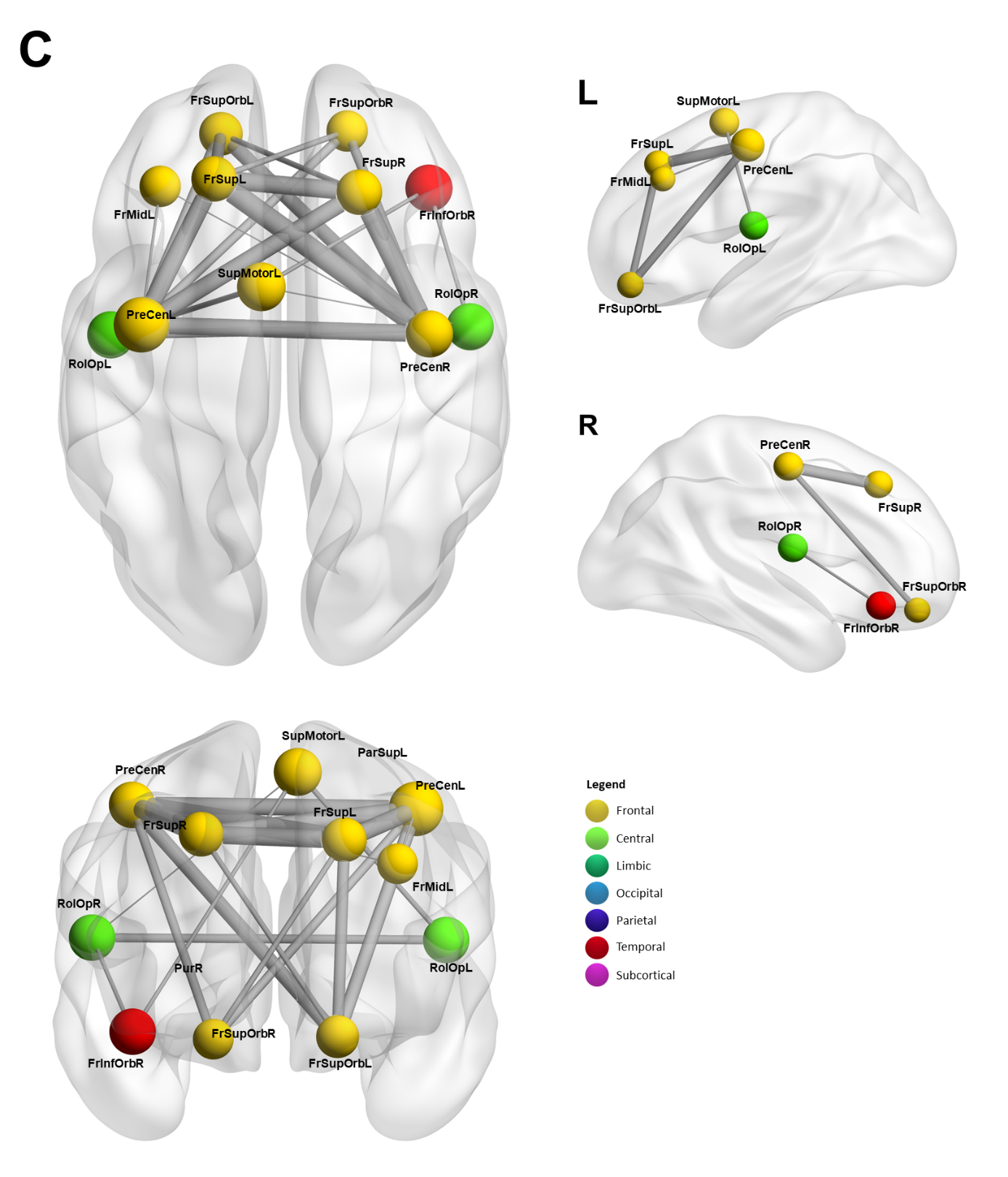

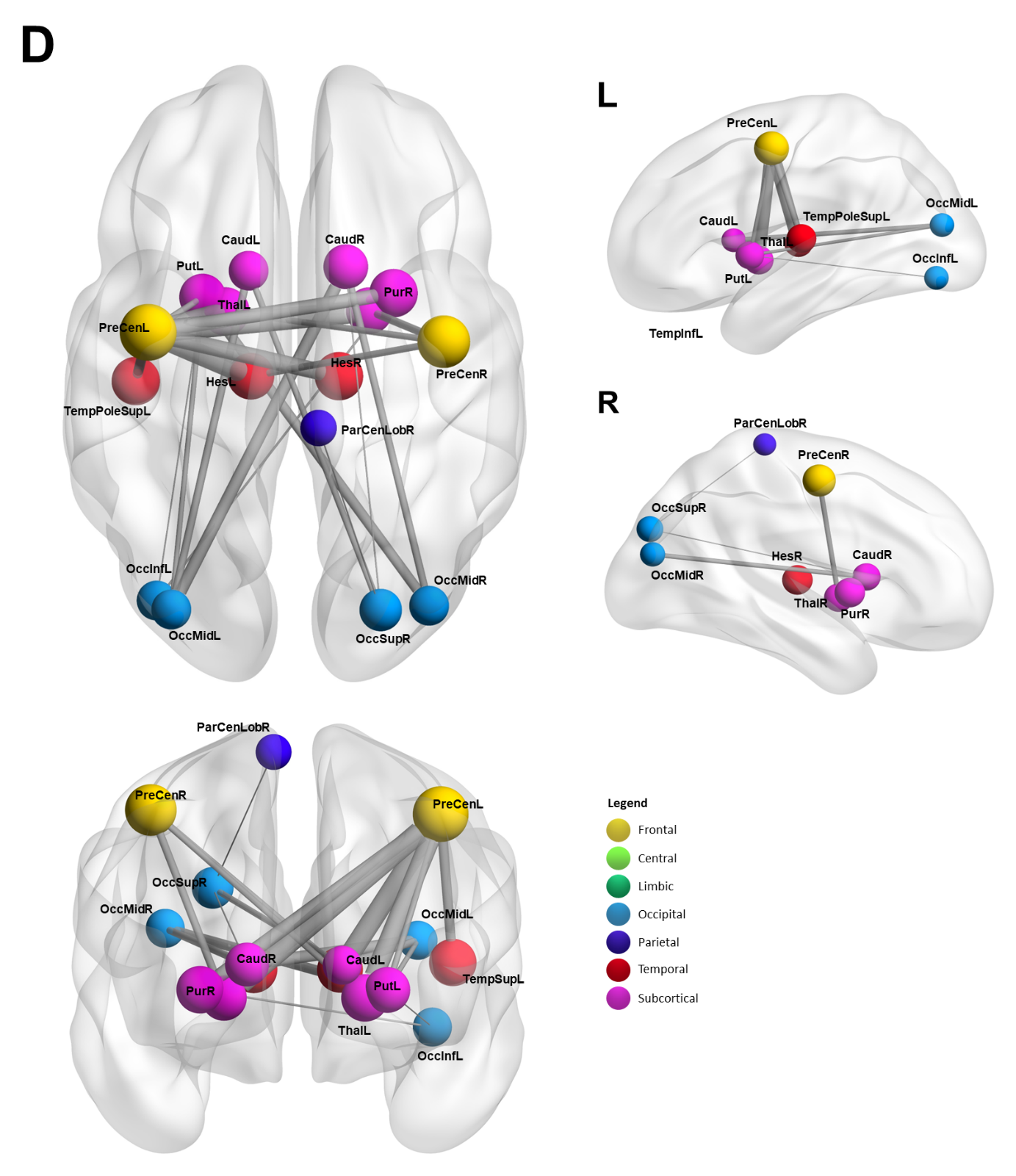
**

**Figure S6.** Learning Curve of the different centres during the adaptation of the inter-centre cross-validation setting. In this setting one centre was used as test set, whereas the other three centres were used as training set. This setting can be strongly affected by sources of uncontrolled variances across scanners and datasets (Abraham et al., 2017; Noble et al., 2017). Therefore, we specifically tested for the effect of transferring subjects from the training set into the test set. Doing so, the test set won’t be fully naïve to uncontrolled variance such as inter-scanner variability, which might benefit classification performance. In each iteration thus, two subjects (1 HC, 1FND) were transferred from the test set to the training set. An increase in classification performance (across accuracy, sensitivity, and specificity could be observed. After the transfer of 16 – 20 subjects, and consequently with a decrease in number of subjects in the test set, the model started overfitting the results.

**
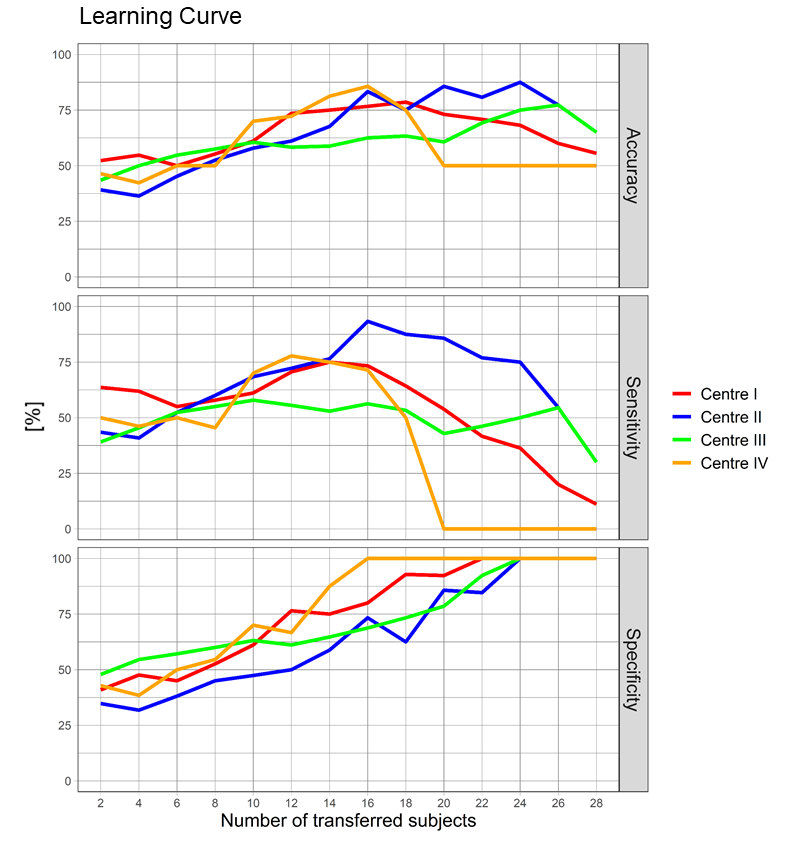
**

**Table S2.** Logistic regression models testing the effects of anxiety (STAI), depression (BDI), psychotropic medication (yes/no), and clinical scores (CGI) taken-together and individually in (A) intra-centre cross-validation, and (B) pooled cross-validation.

| A) Intra-centre cross-validation | | |  |  | |  |  |
| --- | --- | --- | --- | --- | --- | --- | --- |
| **Centre I** | | **Beta coefficients** | **p-value** | **Centre III** | | **Beta coefficients** | **p-value** |
|  | *intercept* | 7.0 | 0.493* |  | *intercept* | -1.081 | 0.753 |
|  | STAI | -0.075 | 0.119 |  | STAI | 0.04 | 0.405 |
|  | BDI | -0.091 | 0.41 |  | BDI | -0.1 | 0.298 |
|  | medication | 1.757 | 0.19 |  | medication | 1.66 | 0.139 |
|  | CGI | -0.928 | 0.122 |  | CGI | -0.321 | 0.673 |
|  | *intercept* | 3.222 | 0.034* |  | *intercept* | 1.174 | 0.303 |
|  | STAI | -0.035 | 0.133 |  | STAI | -0.003 | 0.792 |
|  | *intercept* | 1.246 | 0.04* |  | *intercept* | 0.843 | 0.067 |
|  | BDI | -0.023 | 0.679 |  | BDI | 0.003 | 0.9 |
|  | *intercept* | 1.1 | 0.007** |  | *intercept* | 0.847 | 0.033* |
|  | medication | -0.182 | 0.8 |  | medication | 0.108 | 0.87 |
|  | *intercept* | 1.576 | 0.08 |  | *intercept* | 1.548 | 0.146 |
|  | CGI | -0.434 | 0.298 |  | CGI | -0.415 | 0.481 |
| **Centre II** | |  |  | **Centre IV** | |  |  |
|  | *intercept* | 0.703 | 0.781 |  | *intercept* | 1.107 | 0.649 |
|  | STAI | 0.014 | 0.72 |  | BAI | 0.032 | 0.673 |
|  | BDI | -0.043 | 0.646 |  | BDI | -0.868 | 0.335 |
|  | medication | -1.32 | 0.292 |  | medication | - | - |
|  | CGI | 0.234 | 0.75 |  | CGI | 0.06 | 0.959 |
|  | *intercept* | -0.032 | 0.979 |  | *intercept* | 1.01 | 0.309 |
|  | STAI | 0.015 | 0.4 |  | BAI | 0.0001 | 0.998 |
|  | *intercept* | 0.737 | 0.076 |  | *intercept* | 1.5 | 0.086 |
|  | BDI | 0.039 | 0.381 |  | BDI | -0.054 | 0.418 |
|  | *intercept* | 1.003 | 0.004** |  | *intercept* | - | - |
|  | medication | -0.087 | 0.924 |  | medication | - | - |
|  | *intercept* | 1.39 | 0.126 |  | *intercept* | 0.793 | 0.705 |
|  | CGI | -0.123 | 0.822 |  | CGI | 0.087 | 0.914 |

| B) Pooled cross-validation | | |  |
| --- | --- | --- | --- |
|  |  | **Beta coefficients** | **p-value** |
|  | *intercept* | 0.555 | 0.603 |
|  | STAI | -0.007 | 0.652 |
|  | BDI | 0.007 | 0.856 |
|  | medication | 0.995 | 0.08 |
|  | CGI | 0.049 | 0.87 |
|  | *intercept* | 0.97 | 0.102 |
|  | STAI | -0.002 | 0.809 |
|  | *intercept* | 0.835 | <0.001*** |
|  | BDI | 0.005 | 0.787 |
|  | *intercept* | 0.708 | <0.001*** |
|  | medication | 0.496 | 0.253 |
|  | *intercept* | 0.179 | 0.681 |
|  | CGI | 0.317 | 0.174 |

Abbreviations: STAI: State-Trait Anxiety Inventory, BDI: Beck’s Depression Inventory, CGI: Clinical Global Impression Score. Significance levels with ***p ≤ 0.001, ** p ≤ 0.01, * p ≤ 0.05.

**References**

Richiardi, J., Eryilmaz, H., Schwartz, S., Vuilleumier, P., Van De Ville, D., 2011. Decoding brain states from fMRI connectivity graphs. Neuroimage 56, 616–626. https://doi.org/10.1016/j.neuroimage.2010.05.081

Wegrzyk, J., Kebets, V., Richiardi, J., Galli, S., de Ville, D. Van, Aybek, S., 2018. Identifying motor functional neurological disorder using resting-state functional connectivity. NeuroImage Clin. 17, 163–168. https://doi.org/10.1016/j.nicl.2017.10.012
